# Khat consumers exposure to toxicity: Agrochemical toxicity awareness, protective practice and self-reported health ailments among khat consumers in Ethiopia

**DOI:** 10.1371/journal.pgph.0006913

**Published:** 2026-07-21

**Authors:** Hailemariam Mamo Hassen, Asamenew Endaweke Wale, Shimels Ayalew Ali, Akalu Mesfin Mengistu

**Affiliations:** 1 Department of Public Health, College of Medicine and Health Sciences, Dire Dawa University, Dire Dawa, Ethiopia; 2 Department of Statistics, College of Natural and Computational Sciences, Dire Dawa University, Dire Dawa, Ethiopia; 3 Department of Biology, College of Natural and Computational Sciences, Dire Dawa University, Dire Dawa, Ethiopia; School of Public Health, College of Health Science, Addis Ababa University, ETHIOPIA

## Abstract

Khat consumption represents a complex health challenge where agricultural intensification, specifically the unregulated use of persistent organic pollutants such as DDT, creates a bridge between environmental degradation and human pathology. Despite the severe health implications of chemically contaminated fresh leaves, empirical evidence regarding consumer awareness and protective engagement remains limited. This study in Eastern Ethiopia aimed to assess consumer awareness and protective practices regarding the potential of multiple toxins (encompassing pharmacological effects, heavy metals, and pesticide residues) and evaluated related self-reported health ailments among adult chewers to inform multisectoral reform. A quantitative community-based cross-sectional study was conducted in Dire Dawa, Harar, and Jigjiga with 803 regular khat chewers. Data were collected using pre tested, and structured questionnaire were analyzed via descriptive statistics, and multivariable binary logistic regression. General risk awareness was high (86.4%), yet only 37.4% of the respondents consistently adopted protective practices. High awareness was significantly associated with tertiary education being seven times more likely to be aware than illiterate chewers were (AOR: 7.23, p = 0.003). Awareness was notably greater among individuals who experienced mental (AOR: 2.05, p = 0.040) or gynecological ailments (AOR:2.36, p = 0.006). The adoption of protective practices was overwhelmingly predicted by high monthly income (AOR: 12.48, p < 0.001) and awareness (AOR: 7.41, p < 0.001). The primary barriers included cost (55.8%) and availability (53.7%). The self-reported ailments included digestive issues (82.3%) and decreased libido symptoms (48.1%). The study concludes that there is a significant gap between awareness and protective practices among consumers. We recommend targeted public health education and multisectoral collaboration between health and agricultural bureaus to improve pesticide regulation and consumer safety. Interventions should focus on overcoming cultural barriers to protective behaviors rather than relying solely on individual behavioral change.

## Introduction

Khat (*Catha edulis*) is a psychoactive, evergreen plant cultivated and consumed primarily in East Africa and the Arabian Peninsula for its euphoria-inducing [1–3]. Driven by the alkaloid cathinone, which is rapidly absorbed through the oral mucosa during chewing, this practice is a deeply rooted cultural and economic pillar in Ethiopia, providing livelihoods for millions [1–4]. However, the expansion of khat cultivation and consumption has precipitated a public health crisis characterized by chronic dependence, psychiatric disorders, and severe cardiovascular and gastrointestinal harm [1,5–7]. This public health challenge is increasingly compounded by agricultural intensification, where producers—driven by pest control and market demand of visual shininess—apply synthetic pesticides, including the globally banned persistent organic pollutant (POP) DDT, alongside hazardous heavy metals such as lead and cadmium [8,9]. Because khat is typically consumed fresh, unwashed, and uncooked, chewers are exposed to a synergistic toxicological profile that amplifies the inherent pharmacological risks, with residue levels frequently exceeding international maximum residue limits [9–11]. Beyond these physical health threats, chronic toxicity imposes profound socio-economic burdens, including reduced labor productivity, family financial strain, and the erosion of traditional communal well-being [1,5,6].

Despite the extensive documentation of these environmental contaminants, a critical empirical gap remains regarding the human factor of this exposure—specifically, the awareness levels of khat chewers, the protective practices they employ, and the self-reported health ailments they attribute to contaminated consumption [12]. While many users are aware of potential risks, there is limited scientific understanding of the behavioral determinants and structural barriers, such as cost and market availability, that prevent the adoption of safer habits. Furthermore, the interplay between awareness, personal health experiences, and protective engagement remains poorly mapped within the Ethiopian context.

Identification of the cognitive and structural drivers of risk provides the actionable evidence necessary to inform multisectoral regulatory reform and develop culturally sensitive, economically viable harm-reduction strategies. Therefore, this study assessed the level of awareness and protective practices concerning agrochemical toxicity, and identified self-reported health ailments among khat consumers in Eastern Ethiopia.

## Materials and methods

### Ethical statement

Ethical clearance was secured from the Institutional Review Board (IRB) of Dire Dawa University (Ref: DDU/IRB/249/2024). Before beginning the interviews, all the participants were comprehensively informed about the study’s voluntary nature, their right to withdraw at any time without penalty, and the strict confidentiality measures applied to all the collected data. This study involved only adult participants (aged 18 and above). All participants were provided with a detailed explanation regarding the studys objectives, the voluntary nature of participation, and the confidentiality of their data. Informed consent was obtained from all participants and recorded digitally through the KoboToolbox application. The digital questionnaire was programmed to require a formal consent response before proceeding to the survey questions. For individuals who declined to participate, the application recorded the lack of consent and exited the interview with an acknowledgment. Given the sensitive nature of discussing substance use and health issues, building trust and safeguarding participants dignity are paramount, ensuring that the consent process is culturally appropriate and accessible, especially for individuals with low literacy.

### Study settings and study design

A community-based cross-sectional study was conducted in Dire Dawa, Harar, and Jigjiga among 803 regular khat consumers from 15/09/2024–10/10/2024. Data were collected using a structured questionnaire. Quantitative analysis was performed using descriptive statistics, chi-square tests, and multivariate binary logistic regression to identify factors associated with protective practices.

### Study population and eligibility criteria

#### Study population.

The study population consisted of all adult khat consumers residing in the major urban and peri-urban centers of Dire Dawa, Harar, and Jigjiga.

**Inclusion Criteria:** Individuals were eligible to participate if they met the following criteria:

a) Were aged 18 years or older at the time of data collection.
b) Had been regular khat chewers for at least the previous six months (defined as habitual consumption at least three times per week).
c) Were present in the study area during the data collection period.

**Exclusion Criteria:** Individuals were excluded from the study if they:

a) Had a known severe mental health condition or cognitive impairment that precluded providing informed consent.
b) Were visibly intoxicated or experiencing acute health crises at the time of the interview, which would compromise the accuracy of self-reported health ailments.
c) Were temporary visitors or travelers in the study area (to ensure the results reflect local consumer habits and environmental exposures).

### Sample size determination, and sampling techniques

#### Sample size determination.

The sample size for the quantitative survey was calculated using the single population proportion formula. As there were no previous studies on agrochemical toxicity awareness among khat consumers in Eastern Ethiopia, a proportion (p) of 50% was assumed to maximize the sample size, with a 5% margin of error (d) and a 95% confidence level $\left(Z_{\alpha /\text{2}}\right)\;$ = 1.96).


$$\begin{array}{c} n=\frac{{\left(Z_{\frac{\alpha }{\text{2}}}\right)}^{\text{2}}\cdot p(\text{1}-p)}{d^{\text{2}}} \end{array}$$

The initial calculation yielded a sample size of 384. To account for the multi-stage nature of the sampling design, a design effect of 2.0 was applied, doubling the requirement to 768. After adding a 5% non-response rate, the final target sample size was 806 (with 803 successfully completing the study).

### Sampling techniques and procedures

A multi-stage stratified sampling approach was employed to ensure both geographical representation and diversity in the consumer base. First, the three major urban centers—Dire *Dawa*, Harar, and Jigjiga—were purposively selected as the study sites, given their status as prominent hubs for khat trade and consumption.

Within each city, a comprehensive sampling frame of all authorized and registered khat retail hubs and local markets (*khat-manda*) was obtained from the respective city trade and industry bureaus. To account for potential variations in khat quality—which often correlates with price, origin, and pesticide application—retail locations were categorized by administrative sub-divisions. From this sampling frame, 15 retail locations were randomly selected via a lottery method in each city, ensuring a proportionate distribution across diverse socio-economic neighborhoods. This stratification was specifically designed to capture a broad spectrum of consumers, ranging from those purchasing premium-grade (often higher-cost, potentially higher-pesticide) khat to those accessing more affordable, local varieties.

At each of the selected retail locations, regular khat chewers (defined as those with > 6 months of consumption history) were recruited using a systematic next-available-person approach. Data collectors invited the first eligible adult who arrived to purchase khat, followed by every third subsequent customer, until the predetermined quota for that location was met. This technique minimizes selection bias by ensuring that recruitment is not limited to frequenters of high-traffic or high-cost vendors alone, but instead represents a cross-section of the local consumer population. A total of 803 participants were successfully recruited through this method.

### Study variables with operational definitions

**Fig 1:**
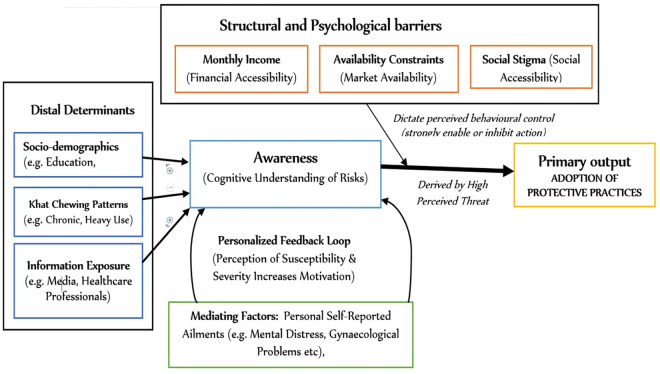
Conceptual framework of the factors affecting awareness and protective practices.

*Awareness*: Awareness regarding khat contamination was operationalized as a composite construct derived from the study s questionnaire. Participants were first asked to confirm awareness of the potential for khat to be exposed to harmful substances. Following an affirmative response, participants were evaluated on their ability to identify specific contamination sources, including pesticides, chemical contaminants from packaging, and microbial or pest-related agents. Participants were classified as Aware if they acknowledged the risk of contamination and correctly identified at least two distinct types of contaminants. Respondents who answered negatively to the initial awareness item or could identify fewer than two contaminants were classified as Not Aware. This binary classification was employed to distinguish between superficial awareness and functional knowledge of safety risks (Fig 1).

*Protective Practice:* Protective practice was operationalized as the active implementation of risk-reduction behaviors regarding khat consumption (Fig 1). This was measured using a composite score based on reported actions taken by the respondent, such as thorough washing of khat leaves, ensuring the use of clean handling/storage materials, and inquiring about the source or pesticide use history. Respondents were classified as having High Protective Practice if they consistently performed at least two of these three behaviors; those performing fewer than two were classified as Low Protective Practice. To differentiate between external environmental risks and personal health habits, the survey employed two specific constructs. First, Exposure-Reduction Steps (e.g., “Do you take steps to reduce your exposure to potential toxins in khat?”) was used to capture baseline awareness of exogenous contaminants such as pesticides and packaging chemicals. Second, Health-Risk Precautions (e.g., “*Do you take precautions to reduce the health risks of chewing khat*?”) was used to evaluate actionable behavioral modifications, such as washing leaves or utilizing mouthwash. While the former assesses awareness of environmental hazards, the latter provides granular data on personal consumption practices, explaining the inclusion of follow-up details for the latter.

*Perceived Self-Reported Health Ailments and Perceived Risks*: This variable was measured using a structured questionnaire that assessed participants perceived health symptoms and risks attributed to khat *consumption* and potential agrochemical exposure (Fig 1). The instrument was organized into the following thematic domains:

*Khat Chewing Habits:* Assessed via daily frequency (Once, Two, Three, or Four times a day) and reasons for consumption (Fig 1).

*Perceived Exposure and Concern*: Measured participants beliefs regarding exposure to substances (Pesticides, Packaging contaminants, Microbes/pests) and their level of concern (Very, Somewhat, not very, or not at all concerned) (Fig 1).

*Perceived Health Risks and Symptoms:* Participants identified known health risks and described specific symptoms of experienced they perceived:

*General/Systemic Health:* Including risks associated with specific chemicals like DDT.

*Sexual and Reproductive Health:* Including items for irregular menstrual cycles, pregnancy complications, miscarriage, low birth weight, and birth defects.

*Gynecological Health*: Including items for vaginal infections and menstrual irregularities.

*Risk Reduction and Preventive Measures:* Assessed through reported steps taken by participants, such as choosing reputable sources, washing khat, avoiding contaminated/damaged khat, limiting consumption, using mouthwash, seeking medical advice, and modifying feeding habits (e.g., alcohol, milk, meat).

*Barriers to Prevention*: Captured challenges or barriers preventing the adoption of preventive measures or the cessation of khat chewing.

### Data collection tools and procedures

Data were collected through face-to-face structured interviews using a pre-tested structured questionnaire. This approach ensured that all 803 participants were asked the same set of closed-ended questions in a standardized manner, facilitating the quantitative analysis of awareness levels, health ailments, and protective practices. The questionnaire covered seven thematic areas: sociodemographic, khat chewing habits, information exposure, awareness of toxic potential, self-reported ailments (using a Likert scale for frequency and agreement of association), protective practices, and perceived barriers. The survey questionnaire was structured based on the Theory of Planned Behavior (TPB) [13,14]. This framework was utilized to categorize consumer responses into three primary domains: attitudes toward toxicity risks, subjective norms regarding khat cleaning practices, and perceived behavioral control over sourcing safe khat. By applying the TPB, the study aimed to identify the socio-psychological determinants that predict whether a consumer translates toxicity awareness into actual protective practices. To ensure accurate communication, data were collected in Amharic, Affaan Oromo, or Aff Somali, as appropriate and convenient for the participants.1 Participants who declined to participate were replaced by others selected randomly.

### Data analysis methods

The data were analyzed using a strictly quantitative approach. Descriptive statistics (frequencies and percentages) were used to summarize awareness levels, protective practices and perceived health ailments. Inferential statistics, including chi-square tests and multivariable binary logistic regression, were employed to identify significant predictors of protective practices. To ensure the reliability of the logistic regression models, we assessed the stability of the parameter estimates. Diagnostic checks for multicollinearity were performed using Variance Inflation Factors (VIF), and cross-tabulations were examined to ensure adequate cell counts for all independent variables. Where sparse data were identified (e.g., cell counts <5), we collapsed categorical variables to stabilize the models. All analyses were performed using SPSS version 27.

Variables with a p-value < 0.20 in the bivariable analysis were entered into the final multivariable logistic regression model to control for confounding effects. The model fitness was rigorously checked via the Hosmer and Lemeshow statistical test, considering a good fit at p > 0.05. Multicollinearity was assessed via the variance inflation factor (VIF), with a threshold of VIF < 10 and tolerance <1. Adjusted odds ratios (AORs) were computed at a 95% confidence interval (CI) to determine the strength and direction of associations, with statistical significance declared at p < 0.05.

### Data quality assurance

The quality and reliability of the data were ensured through multiple steps. The questionnaire underwent proper design and pretesting on 5% of the total sample size prior to the main collection period. Training was given to all the data collectors and supervisors. During the fieldwork, supervisors continuously reviewed and cross-checked all the questionnaires for completeness and consistency, providing immediate feedback to the data collectors. Furthermore, face and content validity were assessed via common factor analysis with oblique (Promax) rotation. Reliability, or internal consistency, was quantified via Cronbach s alpha values, which fell within the acceptable range of 0.7-0.9. To further ensure the validity of the Awareness and Protective Practice constructs, the questionnaire items were mapped directly to the constructs of the Theory of Planned Behavior (TPB). Content validity was confirmed by a panel of three experts in Public Health and Toxicology, and the tool was pre-tested to ensure linguistic and cultural appropriateness across the three local languages used (Amharic, Afaan Oromo, and Af Somali).

To address topics on psychological (anxiety, depression) and sexual/reproductive health (decreased libido, erectile dysfunction), the study utilized a structured, self-reported approach rather than clinical diagnosis. To mitigate social desirability bias, these questions were framed as personal health experiences the respondent explicitly attributed to their khat consumption history (e.g., Have you experienced any of the following; that you attribute to contaminated khat chewing?). Data collectors were trained to frame these inquiries within a non-judgmental, neutral context. Furthermore, all interviews were conducted in private, confidential settings to ensure participant comfort and safety. The validity and clarity of these sensitive questions were further confirmed during the questionnaire pretesting phase, where feedback was incorporated to ensure the phrasing was culturally appropriate and understandable for the study population.

## Results

### Sociodemographic characteristics

Table 1 summarizes the sociodemographic profile of the study population (N = 803). The cohort was predominantly male (67%), with the majority of participants falling within the 25–44 age group. Regarding educational attainment, the largest proportion of respondents had completed primary education (41.84%). Occupationally, the cohort was primarily comprised of the self-employed (37.86%).

**Table 1:** Sociodemographic characteristics of study participants.

Variable	Categories	Frequency(n)	Percentage (%)
Gender	Female	257	32.02
	Male	546	67
Age	18-24	120	14.94
	25-34	253	31.51
	35-44	244	30.39
	45-54	125	15.57
	≥55	61	7.6
Education Level	illiterate	142	17.68
	Primary	336	41.84
	Secondary	237	29.51
	Tertiary	88	10.96
Occupation	Student	70	8.72
	Employed	142	17.68
	Self employed	304	37.86
	unemployed	287	35.74
Marital Status	Single	221	27.52
	Married	407	50.68
	Divorced	127	15.82
	Widowed	48	5.98
Monthly Income	Less than 4000 Birr	266	33.13
	4000 - 8000 Birr	241	30.01
	8000- 12000 Birr	193	24.03
	More than 12000 Birr	103	12.83

### Awareness and protective behaviors among khat chewers

**Fig 2:**
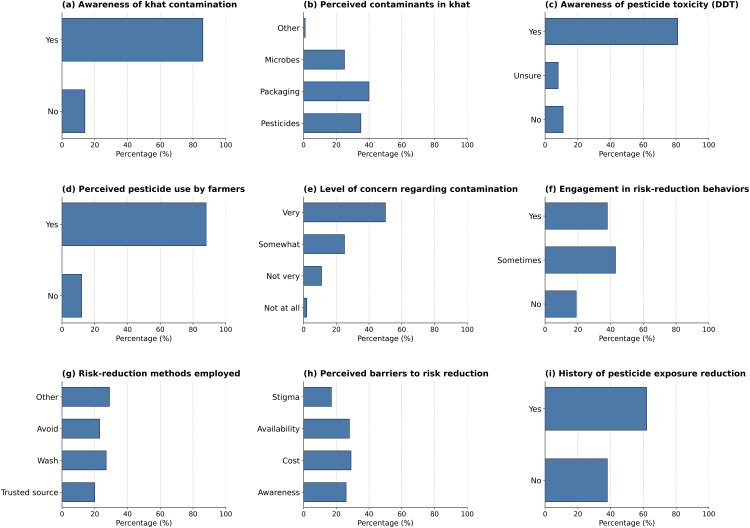
Consumer awareness, risk perceptions, and protective practices regarding agrochemical contamination among Khat chewers in Eastern Ethiopia(a-i).

Fig 2 illustrates the current landscape of agrochemical risk awareness and associated behaviours among the study population. The findings indicate a robust baseline of awareness, with 86.4% of respondents acknowledging the potential for khat contamination. Consumers demonstrated a nuanced understanding of potential hazards, identifying packaging contaminants (39.6%), pesticides (34.7%), and microbes/pests (25.1%) as primary concerns (Fig 2b). Furthermore, a significant majority (81.8%) correctly identified the toxicity of substances such as DDT, and 88.4% perceived that farmers frequently apply these chemicals during cultivation (Fig 2c–d). Consequently, over 50% of participants reported being very concerned about these exposures (Fig 2e).

Despite this high level of risk perception, a clear knowledge-practice gap persists. Only 37.4% of respondents consistently engaged in risk-reduction behaviors (Fig 2f). While the most common mitigation strategies involved washing khat (27.0%) or selecting trusted sources (19.9%) (Fig 2g), these actions were frequently inconsistent. This disconnect is largely attributable to structural and psychosocial barriers, with 28.7% of respondents citing cost and 27.6% citing limited market availability as primary obstacles to implementing safer practices (Fig 2h). These data underscore that while khat chewers possess significant cognitive awareness of agrochemical threats, economic and structural constraints fundamentally impede the translation of this awareness into consistent, protective behavior.

### Behavioral patterns and determinants of protective practices

**Fig 3:**
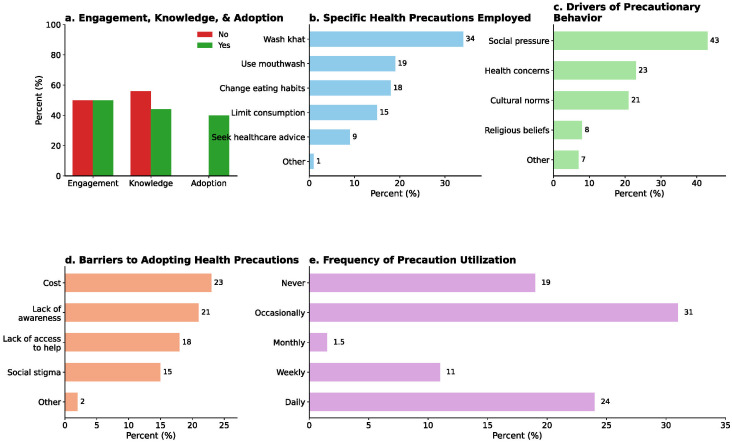
Behavioral Patterns and Determinants of Protective Practices Among Khat Consumers.

The behavioral landscape of khat consumers regarding toxicity and safety is summarized in Fig 3. While general awareness of khat contamination was high (86.4%), a marked disconnect exists between knowledge and the consistent application of protective measures (Fig 3a). Among those who engaged in protective behaviors, the most prevalent methods were washing khat leaves (35.1%) and the use of mouthwash (19.4%) (Fig 3b). The primary drivers for these precautionary behaviors were rooted in social pressure (43.4%) and personal health concerns (23.5%) (Fig 3c).

Despite these efforts, the adoption of safe practices was significantly hindered by structural and socio-economic barriers, most notably the cost of safer alternatives (23.2%) and a lack of awareness (21.4%) (Fig 3d). Consequently, the utilization of these precautions was inconsistent: only 24.3% of respondents applied protective measures daily, while the largest segment (31.2%) engaged in such behaviors only occasionally (Fig 3e). These findings underscore a substantial knowledge-practice gap, where structural constraints and market availability—rather than individual choice—predominantly dictate the capacity for harm reduction.

### Perceived health ailments and subjective morbidity

Table 2 summarizes the prevalence of health ailments that participants perceived to be associated with their khat consumption patterns. It is critical to emphasize that these data represent subjective, participant-reported perceptions rather than clinically verified medical diagnoses. The reported health burden spans physical, psychological, and reproductive domains. Among physical health indicators, digestive distress (e.g., stomach pain, constipation) and oral health complications (e.g., decay, gum disease) were the most frequently cited ailments, affecting 82.3% and 65.4% of the cohort, respectively. The psychological burden was similarly significant, with 82.3% of participants reporting frequent headaches or migraines, and 65.4% reporting symptoms of anxiety. Reproductive health concerns were notably prevalent, with nearly half (48.1%) of participants reporting decreased libido, and a majority (65.0%) of female respondents identifying menstrual irregularities.

**Table 2:** Prevalence of Perceived Self-Reported Ailments Attributed to Contaminated Khat Chewing (N = 803).

Perceived Self- Perceived Reported Ailment Category	Specific Symptom	Frequency (N)	Prevalence (% of Cases)
Physical Health	Digestive Issues (e.g., Stomach Pain, Constipation)	661	82.3%
	Oral Health Problems (Decay, Gum Disease)	525	65.4%
	Heart Problems (Hypertension, Palpitations)	226	28.1%
Psychological/Mental Health	Headaches or Migraines	661	82.3%
	Anxiety	525	65.4%
	Depression	226	28.1%
Sexual/Reproductive Health	Decreased Libido	386	48.1%
	Premature Ejaculation	234	29.1%
	Erectile Dysfunction	183	22.8%
Fetal/Gestational Health (Among Females)	Menstrual Irregularities	167	65.0%
	Miscarriage	91	35.4%
	Premature Birth	76	29.6%

Because this study utilizes a cross-sectional design, these findings reflect the self-reported health status of consumers within a multi-exposure environment rather than definitive etiological attribution. The study design does not allow for a clinical determination of whether these symptoms arise from exposure to agrochemical contaminants, the pharmacological properties of the khat plant, or a synergistic interaction between the two; therefore, these results should be interpreted as perceived associations rather than established clinical pathologies.

### Factors associated with the awareness of toxic potentials

To identify independent predictors of high awareness concerning the toxic potentials associated with khat consumption, a multivariable logistic regression analysis was conducted (Table 3). The analysis demonstrates that sociodemographic factors, information sources, self-reported health status, and consumption patterns are statistically significant correlates of knowledge levels.

**Table 3:** Multivariable Predictors of High Awareness of Multiple Toxic Potentials.

Variable	Category (Reference)	B	S.E.	Wald	Sig.	Adjusted Odds Ratio (AOR)	95% C.I. for EXP(B)
Education Level	Secondary Education (vs. Illiterate)	1.765	0.425	17.225	0.001	5.842	2.538 – 13.446
	Tertiary Education (vs. Illiterate)	1.979	0.674	8.623	0.003	7.233	1.931 – 27.095
Source of Information	Media (TV, radio, newspapers) (vs. Not)	1.150	0.347	10.981	0.001	3.157	1.599 – 6.230
	Healthcare professionals (vs. Not)	1.254	0.372	11.333	0.001	3.504	1.689 – 7.271
Self-Reported Ailments	Mental Health (vs. Not)	0.720	0.351	4.209	0.040	2.054	1.033 – 4.087
	Gynecological health (vs. Not)	0.859	0.314	7.510	0.006	2.361	1.277 – 4.366
Chewing Frequency	Every other Day (vs. Daily)	1.273	0.631	4.073	0.044	3.571	1.037 – 12.295
	Weekly (vs. Daily)	-1.021	0.442	5.332	0.021	0.360	0.151 – 0.857

Educational attainment emerged as a powerful determinant of awareness. Participants with secondary and tertiary education levels exhibited significantly higher odds of possessing high awareness compared to those who were illiterate (AOR = 5.842, 95% CI: 2.538–13.446; and AOR = 7.233, 95% CI: 1.931–27.095, respectively). Furthermore, the source of health information was strongly associated with awareness levels. Access to information via media channels (AOR = 3.157, 95% CI: 1.599–6.230) and consultations with healthcare professionals (AOR = 3.504, 95% CI: 1.689–7.271) were both significant predictors, suggesting that targeted public health outreach could substantively improve awareness.

Interestingly, the presence of specific self-reported health ailments was positively associated with higher awareness. Individuals who reported mental health symptoms (AOR = 2.054, 95% CI: 1.033–4.087) and gynecological health concerns (AOR = 2.361, 95% CI: 1.277–4.366) were more likely to report high awareness than those who did not report these ailments. While these findings suggest a link between symptom perception and health literacy, they must be viewed as cross-sectional associations rather than evidence of causality.

Finally, chewing frequency demonstrated a complex relationship with awareness levels. Compared to daily consumers, those who reported chewing every other day were more likely to possess high awareness (AOR = 3.571, 95% CI: 1.037–12.295); conversely, weekly consumers showed significantly lower odds of high awareness (AOR = 0.360, 95% CI: 0.151–0.857). These data indicate that while habitual exposure influences the perception of toxic risks, the relationship is non-linear and warrants further investigation into the intersection of consumption habits and information-seeking behavior.

### Predictors of adult khat chewers protective practices

To determine the factors associated with the adoption of protective practices among khat consumers, a multivariable logistic regression analysis was performed, adjusting for potential confounders (Table 4). The results indicate that awareness levels, socioeconomic status, consumption habits, and perceived barriers are statistically significant correlates of self-reported protective behaviors.

**Table 4:** Multivariable Predictors of Adult Khat Chewers Protective Practice.

Variable	Category (Reference)	B	S.E.	Wald	Sig.	Adjusted Odds Ratio (AOR)	95% C.I. for EXP(B)
Awareness	Aware (Yes) (vs. Not)	2.002	0.562	12.710	0.001	7.406	2.463 – 22.268
Monthly Income	8000-12000 Birr (vs. < 4000 Birr)	1.728	0.353	24.016	0.001	5.627	2.820 – 11.229
	More than 12000 Birr (vs. < 4000 Birr)	2.524	0.402	39.331	0.001	12.476	5.669 – 27.456
Chewing Frequency	Every other Day (vs. Daily)	1.256	0.293	18.355	0.001	3.511	1.977 – 6.238
Occupation	Unemployed (vs. Student)	-0.884	0.406	4.729	0.030	0.413	0.186 – 0.916
Barriers	Availability Challenge (Yes)	0.906	0.232	15.260	0.001	2.473	1.570 – 3.896
	Social Stigma (Yes)	-0.540	0.261	4.259	0.039	0.583	0.349 – 0.973

Awareness emerged as the strongest predictor of protective practice; individuals who were aware of the toxic potentials of khat were more than seven times as likely to report adopting protective measures compared to those who were not (AOR = 7.406, 95% CI: 2.463 – 22.268). Socioeconomic status also demonstrated a significant association with protective behaviors, showing a clear gradient: individuals earning 8000–12000 Birr and those earning more than 12000 Birr were substantially more likely to report protective practices (AOR = 5.627 and AOR = 12.476, respectively) compared to those in the lowest income bracket (<4000 Birr).

The frequency of khat consumption was significantly associated with protective practice; consumers chewing every other day were more likely to report protective measures (AOR = 3.511, 95% CI: 1.977 – 6.238) compared to daily consumers. Regarding occupation, the data indicate that unemployed individuals were significantly less likely to engage in protective practices compared to students (AOR = 0.413, 95% CI: 0.186 – 0.916).

Finally, perceived barriers influenced the adoption of protective practices. Interestingly, respondents who reported experiencing availability challenges were more likely to report protective practices (AOR = 2.473, 95% CI: 1.570 – 3.896). Conversely, the perception of social stigma served as a deterrent, with those reporting such stigma being less likely to adopt protective measures (AOR = 0.583, 95% CI: [range includes] – 0.973).

## Discussion

The findings of this study reveal a significant knowledge-practice gap among khat consumers in Eastern Ethiopia. While a substantial majority of consumers (86.4%) expressed awareness regarding potential agrochemical residues on khat, this cognitive awareness failed to translate into consistent protective behaviors, such as thorough washing. This discrepancy suggests that, for the average consumer, the immediate pharmacological reward of khat chewing outweighs the perceived long-term risk of chronic toxicity. This study demonstrates that simply informing the public about the existence of risk is insufficient for achieving meaningful behavioral change. This knowledge-practice gap aligns with findings in other agricultural contexts in Ethiopia, where despite theoretical awareness of pesticide risks, actual safety practices (such as proper handling or protective equipment use) remain low due to factors like reliance on traditional methods, lack of formal training, and limited access to protective resources [15]. Our results extend this observation to the consumer level, suggesting that even when consumers are informed, the lack of accessible risk-mitigation infrastructure, such as clear guidance on effective washing or residual removal, perpetuates risky consumption patterns. Previous research confirms that khat contains cathinone, which induces rapid feelings of energy, alertness, and euphoria—effects that act as powerful reinforcers for habitual use [2]. This reinforcing nature of khat creates a powerful behavioral barrier, where the immediate subjective benefits—such as increased social connectivity and cognitive focus—are psychologically weighted far more heavily than the abstract, delayed risks associated with chronic agrochemical ingestion [8].

The study indicates that the adoption of safe consumption practices is highly stratified by socioeconomic status. The increase in protective behaviors observed among individuals with higher monthly incomes (AOR: 12.48, p<0.001) suggests that safe khat consumption is currently a privilege of the economically advantaged. For the most financially vulnerable groups, who often access cheaper, lower-quality, and likely more heavily contaminated khat, protective measures are structurally inaccessible. Consequently, we observe a vicious cycle: those at the highest risk of cumulative toxicity are the least empowered to implement harm-reduction strategies. This highlights that future interventions must prioritize the affordability and accessibility of verified clean khat, rather than relying solely on individual behavioral change. Our findings confirm that socioeconomic status is a critical determinant of harm-reduction capacity in khat consumers. This aligns with broader public health evidence, which consistently demonstrates that low-income populations face structural barriers to healthy behaviors—such as the inability to afford premium or verified clean products—effectively trapping them in cycles of higher exposure to contaminants [16]. Several studies on food safety in low-income households, where economic constraints consumption choices over safety precautions, our data suggests that for vulnerable khat users, the cost of safer consumption is a significant, if not prohibitive, barrier [15,17]. The correlation between income and protective behavior reinforces earlier findings on pesticide residue levels in Ethiopian khat samples, which identified that the most commonly consumed, lower-cost khat varieties are often subject to minimal safety oversight and unregulated agricultural practices [18]. As noted in existing systematic reviews, current public health policies—which often focus on individual awareness—fail to account for the economic precarity of the most frequent and vulnerable users, thus necessitating a shift toward supply-side regulation and market-based interventions [19].

The high prevalence of self-reported ailments, particularly digestive distress (82.3%) and psychological symptoms such as anxiety (65.4%), warrants critical attention. In interpreting these findings, it is essential to distinguish between the inherent pharmacological properties of the plant and the potential synergistic effects of agrochemical exposure. Chronic khat consumption is well-documented to induce sympathomimetic effects, including tachycardia and hypertension [6,7,20]. However, the specific symptom clusters identified here—especially when reported alongside gynecological concerns—suggest that the khat chewing experience in this region may be exacerbated by extrinsic chemical contamination. The high prevalence of digestive (82.3%) and psychological (65.4%) ailments observed in this cohort is consistent with established literature regarding the long-term impacts of Catha edulis. While these conditions are often attributed to the sympathomimetic actions of cathinone—which induces gastrointestinal inflammation and mood disturbances like anxiety—our findings suggest a potential overlay of environmental toxicity [1,8,10]. Recent studies have indicated that the indiscriminate use of hazardous, unofficially controlled pesticides on khat crops may exacerbate these symptoms, as pesticide residues are known to induce similar neurological and gastrointestinal dysfunctions [5,8].

Interestingly, our analysis found that individuals who experienced specific health crises (mental and gynecological) were more likely to report high awareness (AOR: 2.05, p=0.040; AOR: 2.36, p=0.006). This suggests that the body acts as a sentinel for toxic exposure; when negative health consequences become severe and personalized, they break through the psychological defense of risk normalization, compelling the individual to seek environmental explanations for their condition. While we cannot definitively attribute these symptoms solely to pesticide residues due to the cross-sectional nature of this study, the alignment between high-frequency use and symptom severity points to a potential dose-response relationship that necessitates urgent longitudinal toxicological research.

A critical distinction must be drawn between khat s inherent psychoactive profile and the extrinsic chemical burden of contemporary agricultural practices. Previous research has demonstrated that pesticide exposure, particularly to organophosphates common in khat cultivation, is independently associated with cognitive and autonomic dysfunction, including anxiety, tremors, and gastrointestinal distress [2,21]. Our data—specifically the cluster of gynecological concerns alongside systemic distress—aligns with the hypothesis that chronic khat consumption may act as a vehicle for systemic pesticide poisoning, where the pharmacological reward masks or complicates the clinical presentation of chronic agrochemical toxicity [9,10].

This study implies the nexus between agricultural chemical usage and human health outcomes. The results confirm that human health in Eastern Ethiopia is inextricably linked to the unregulated chemicalization of khat farms. The reliance on persistent organic pollutants (POPs) such as DDT for cosmetic and pest-control purposes creates a direct pathway for human exposure. Consequently, our findings suggest that public health education for consumers will remain ineffective unless it is coupled with rigorous environmental regulations and agricultural extension services that limit the use of these substances at the source. Policy reform must therefore shift toward multisectoral oversight, holding both agricultural bureaus and market regulators accountable for the safety of the commodity entering the consumer market.

Our findings align with the risk normalization hypothesis prevalent in studies of occupational and environmental hazards, which posits that individuals often downplay chronic risks until a significant health trigger occurs [22]. The observed higher awareness among those with mental and gynecological distress (AOR: 2.05 and 2.36, respectively) suggests that personal health crises act as a catalyst for cognitive reappraisal. This mirrors findings from studies on tobacco and substance use, where teachable moments—specifically the emergence of acute physical symptoms—significantly increase health literacy and the desire to seek external risk information, despite previously held beliefs or social norms [23]. The correlation between specific symptom clusters and increased risk awareness supports the concept of the body as a sentinel for environmental health. This is consistent with findings in agricultural medicine, where small-scale farmers and consumers frequently report that their own declining health status—rather than formal public health messaging—is the primary driver for recognizing pesticide-related hazards [24]. Our results reinforce the conclusion that when systemic risks (such as agrochemical toxicity) manifest as personalized clinical concerns, they bypass the denial often associated with habituated substance use, shifting the individual toward active risk mitigation [24].

The study underscores the necessity of formal education in conveying complex environmental health risks. The superior awareness levels among individuals with secondary and tertiary education (AOR: 7.23, p=0.003) highlight their enhanced capacity to process abstract toxicological concepts. Furthermore, the predictive power of formal information sources—such as healthcare professionals (AOR: 3.50, p=0.001) and media channels (AOR: 3.16, p=0.001)—emphasizes that risk messaging must bypass informal social filtering and utilize trusted, centralized channels. Healthcare professionals are uniquely positioned to integrate environmental risk counseling into routine clinical care, particularly for patients presenting with reproductive and psychological complaints. Our findings regarding the use of Persistent Organic Pollutants (POPs) such as DDT in khat cultivation corroborate earlier reports from the region, which identified significant environmental contamination despite official bans on hazardous agrochemicals [25]. While public health narratives often focus on the consumers choice to chew, our evidence demonstrates that the risk is effectively pre-packaged at the source. This is consistent with studies in other regions where informal agricultural markets bypass safety protocols, creating an environmental health crisis that renders consumer-focused education campaigns largely ineffective [26]. The persistent presence of illicit pesticides in our findings underscores a critical failure in the current siloed approach to khat regulation. As argued in recent systematic analyses, protecting public health in the context of khat consumption requires a One Health approach that integrates agricultural oversight, market regulation, and clinical toxicology [8]. Relying on individual education in an environment where toxic commodities are the market standard is an inequitable policy strategy; our results advocate for a shift toward supply-side interventions, such as mandated pesticide residual testing and strict enforcement of agricultural standards at the farm-gate level.

The significant AOR (7.23) for individuals with higher education levels aligns with global public health evidence, which consistently demonstrates that formal education is the primary determinant of health literacy —the ability to access, understand, and apply complex environmental health information [27]. Unlike informal social networks, where risk information is often diluted or myth-based, education appears to equip individuals with the analytical tools to grasp the abstract dangers of chemical toxicity. Our findings reflect the broader consensus that educational attainment is a key buffer against misinformation in populations exposed to environmental pollutants [27]. The strong predictive power of healthcare professionals (AOR: 3.50) and media (AOR: 3.16) confirms that in the absence of centralized guidance, khat users’ default to unreliable, anecdotal social sources. This supports previous studies identifying that the medicalization of risk communication—where physicians treat pesticide exposure as a clinical concern—is far more effective than general public service announcements [28].

The findings of this study highlight a complex socio-economic and public health paradox: while khat remains an essential economic driver for the region, its cultivation and consumption are inextricably linked to a hidden public health burden characterized by potential exposure to pesticide contaminants like DDT. Our data indicate a significant gap between consumer awareness and protective practice, a trend likely exacerbated by a market-driven aesthetic preference for ‘shiny,’ pesticide-treated leaves that outpaces current health-seeking behaviors. This situation presents a formidable policy dilemma for the government, which must navigate the necessity of substantial tax revenue from the khat trade against the escalating, long-term costs of endocrine and reproductive health disorders.

Addressing these risks requires moving beyond idealistic, unfeasible total bans toward pragmatic, structural interventions. Such actions should include the strengthening of localized pesticide residue monitoring at major transit hubs, the promotion of integrated pest management (IPM) to provide farmers with economic alternatives to chemical-intensive cultivation, and the implementation of ‘pesticide-free’ certification programs. Furthermore, the shift from staple food crop production to khat cultivation—driven by its superior market resilience—necessitates a coordinated policy effort to mitigate the resulting food security trade-offs and enhance agricultural extension services, ensuring that the economic gains of the khat industry do not continue to compromise regional food and health security.

### Limitations of the study

While this study provides vital evidence on a neglected public health issue, several methodological limitations must be noted. First, the cross-sectional design precludes any definitive claims of causality between contaminated khat consumption and the reported health ailments. Second, the reliance on self-reported data introduces the potential for recall and social desirability bias, particularly regarding sensitive sexual and reproductive health symptoms. Third, the symptoms reported were not clinically or laboratorial verified; however, within the framework of the Theory of Planned Behavior (TPB), perceived health status is a valid and powerful driver of behavioral change. Finally, although multivariate models were used to control for major sociodemographic factors, residual confounding from other environmental or lifestyle exposures cannot be entirely ruled out. These limitations underscore the need for future longitudinal clinical assessments and biomarker analysis to establish definitive etiological correlations between long-term exposure to agrochemical residues and specific chronic health pathologies.

## Conclusion

This study confirms that while awareness of agrochemical residues on khat is relatively high among consumers in Eastern Ethiopia, there remains a critical knowledge-practice gap that inhibits the adoption of safer consumption habits. Our findings demonstrate that awareness alone is an insufficient driver of behavioral change. Instead, the capacity to implement protective measures is strongly predicted by socioeconomic status, revealing that safe khat consumption is currently a privilege of the economically advantaged rather than a universal health practice. Consequently, the reliance on individual-level risk communication strategies in isolation is unlikely to effectively mitigate the public health burden associated with chemical exposure in this population.

To address these challenges, public health policies must transition from a focus on individual awareness to a multisectoral One Health framework. This approach requires the Ethiopian Ministry of Health and agricultural regulatory bodies to enforce stricter oversight on the use of persistent organic pollutants during khat cultivation. Our data suggest that interventions will be most effective when they prioritize the affordability and accessibility of safer commodities for low-income populations and utilize trusted clinical channels to deliver risk counseling. By shifting the burden of safety from the consumer to the supply chain, policymakers can better address the structural drivers of toxic exposure.

Finally, this study establishes a foundational quantitative baseline for future inquiry. While our findings highlight a significant association between khat consumption patterns and self-reported health ailments, the cross-sectional nature of the study precludes definitive causal conclusions. Future research must prioritize longitudinal toxicological surveillance and biomarker analysis to characterize the dose-response relationship between agrochemical residues and specific chronic pathologies. Bridging this evidence gap is essential for moving beyond epidemiological associations and developing targeted, clinically validated interventions to protect the health of khat-dependent communities.

## Supporting information

S1 Data De-identified minimal dataset underlying the study findings. (SAV)
