# Supplementary material for: Khat consumers exposure to toxicity: Agrochemical toxicity awareness, protective practice and self-reported health ailments among khat consumers in Ethiopia
Source: PLOS Glob Public Health. 2026 Jul 21;6(7):e0006913. doi: 10.1371/journal.pgph.0006913 (PMC13387539; doi:10.1371/journal.pgph.0006913)
Supplement: Attachment — Submitted filename: Response to editor comments.pdf [file pgph.0006913.s003.pdf]

Date 10/03/2026

Dear Jan Louis Adajo Non, Editor, PLOS Global Public Health,

Thank you for the opportunity to provide the additional information required for our manuscript. We have addressed the editorial requests as follows:

1. **Recruitment Period:** We have updated the Methods section to state that the participant recruitment and data collection period took place from **15/09/2024 to 10/10/2024**.
2. **Participant Consent:** We have clarified the consent process in the Ethics statement. All participants were adults, and informed consent was obtained and recorded digitally via the KoboToolbox application. Participants who did not provide consent were excluded with an acknowledgement, a process documented in the supplementary files.

We have included the revised text for the manuscript below.

Kind regards,

Dr. Hailemariam Mamo Hassen

Corresponding Author
