# Supplementary material for: Khat consumers exposure to toxicity: Agrochemical toxicity awareness, protective practice and self-reported health ailments among khat consumers in Ethiopia
Source: PLOS Glob Public Health. 2026 Jul 21;6(7):e0006913. doi: 10.1371/journal.pgph.0006913 (PMC13387539; doi:10.1371/journal.pgph.0006913)
Supplement: Attachment — Submitted filename: Point by point responses .pdf [file pgph.0006913.s005.pdf]

Point by point responses

Khat Consumers Exposure to Toxicity: Agrochemical Toxicity Awareness, Protective Practice and Self-Reported Health Ailments Among Khat Consumers in Ethiopia-PLOS Global Public Health - PGPH-D-26-00831

Editor comments and authors response

Comment1. Refer Guidelines for resubmitting your figure files are available below the reviewer comments at the end of this letter.

Authors response: Thank you. We have carefully reviewed the guidelines for resubmitting

Comment 2. Potential Copyright Issues:

Figure 1: please (a) provide a direct link to the base layer of the map

Authors response: We appreciate this feedback regarding copyright compliance. To ensure strict adherence to the journal's licensing policies, we have removed Figure 1 entirely. In its place, we have provided a detailed text description of the study area in the [Insert Section Name] section, which conveys the necessary geographical context for the reader without requiring copyrighted map assets.

Figure 2: Please confirm whether you drew the images / clip-art within the figure panels by hand.

Authors response: Yes! the figure is made by hand. It is now modified and relocated from discussion section to the methods section per the comment and renamed as Figure 1 in the revised manuscript.

<u>Point by point responses for the reviewers comments</u>

Reviewer1

<u>**Comments**</u>**:**	**Authors response Response**
General Comment 1: - This manuscript addresses an important public health issue in Ethiopia. However, the study suffers from serious methodological flaws that compromise the validity and clarity of the findings.	We sincerely thank the reviewer for their critical evaluation and for recognizing the public health significance of this research. We acknowledge that the initial manuscript lacked the necessary methodological clarity, specifically regarding our study design. To address these serious concerns, we have undertaken a comprehensive revision of the manuscript to ensure that the methodology is coherent, rigorous, and accurately reported.<br>*Specifically, we have:*<br>Refined the Study Design: We have fully restructured the manuscript to represent a purely quantitative cross-sectional study, removing previous references to mixed methods that were not adequately supported by our data.<br>Clarified Conceptual Scope: We have removed the One Health framework to ensure the scope of our conclusions strictly aligns with our quantitative cross-sectional findings.<br>Improved Structural Alignment: We have condensed the manuscript and aligned it with IMRaD formatting to improve logical flow and resolve the clarity issues identified by the reviewer.
- General Comment 2: The design is poorly articulated (cross-sectional vs. mixed methods), the application of the One Health lens is unjustified, the data collection and analysis procedures are inconsistently described, and the results are presented in a confusing manner with excessive tables and unclear distinctions between quantitative and qualitative components.	We appreciate the reviewer's detailed feedback, which has been instrumental in refining the study's clarity and focus. We acknowledge that the initial manuscript suffered from methodological inconsistencies. In the revised manuscript, we have implemented the following comprehensive changes to resolve these issues:<br>1. Methodological Alignment: We have explicitly redefined the study design as a quantitative cross-sectional study. We have removed all prior references to mixed methods and One Health frameworks, as these were not adequately supported by our data.<br>2. Procedures & Consistency: We have significantly revised the Methods section to ensure that data collection and analysis procedures are described with technical precision, aligning strictly with a quantitative, observational design.<br>3. Restructuring for Clarity: To address the concerns regarding the results presentation, we have consolidated our data, removed redundant tables, and eliminated qualitative components. The manuscript has been restructured according to the IMRaD (Introduction, Methods, Results, and Discussion) format, ensuring a unified narrative and a clear, logical flow throughout the results section

General Comment 3: The discussion does not adequately interpret the findings in light of the methodological limitations. A major revision is required to clarify the study design, align methods with objectives, streamline results, and provide a coherent discussion.	We thank the reviewer for this essential critique. We agree that the original manuscript lacked the necessary critical depth in the Discussion section and suffered from structural incoherence. In response, we have performed a major revision to realign the entire manuscript:<br>Clarification of Design & Objectives: We have re-aligned our research objectives to strictly reflect the quantitative, cross-sectional design of the study, ensuring there is no ambiguity regarding our methodological approach.<br>Re-structured Discussion: The Discussion has been completely rewritten to provide a coherent narrative. We have integrated a new subsection that explicitly interprets our findings in the context of the study's methodological limitations (e.g., self-reported data and cross-sectional constraints), fulfilling the reviewer's requirement for a more nuanced analysis.<br>Streamlined Results: As part of this revision, we have condensed the Results section to remove redundant tables and focus exclusively on data that directly supports our refined research objectives, ensuring a logical flow from hypothesis to evidence.
Specific comments are outlined below	
Comment 1: Abstract part: - Background: well written, the problem and gap are indicated. However, the objectives need to be rewritten because the word explored cannot be used for a quantitative study. Instead, use assess since the study design is a cross-sectional study.	We sincerely thank the reviewer for this precise observation. We agree that terminology must strictly align with the methodological framework of the study. Accordingly, we have updated the objective in the Abstract to use the term assess, ensuring it accurately reflects our quantitative, cross-sectional design
Comment 2: Abstract part: - Methods part: there is some confusion.<br>Utilizing a One Health lens, a cross-sectional study was conducted in Dire Dawa, Harar, and Jigjiga with 803 regular khat chewers. Data from semi structured interviews were analyzed via descriptive statistics, chi-square tests, and multivariate binary logistic regression for quantitative components, whereas qualitative data were subjected to inductive thematic analysis to provide rich contextual detail regarding lived, experiences. This paragraph is very confused.	We appreciate the reviewer's observation regarding the clarity of our study design. We apologize for any ambiguity in our initial draft. We confirm that this study employed a quantitative, community-based cross-sectional study design. The abstract has been revised to remove references to mixed-methods or One Health approaches, ensuring it accurately reflects our quantitative methodology, which utilized structured questionnaires to assess prevalence, associated factors, and predictors of protective practices among khat consumers through descriptive statistics, and multivariable binary logistic regression.
Comment 3: Abstract part: - i. The study design: is it only a cross-sectional study or a mixed method? If mixed, what type of mixed (sequential, concurrent, etc.)?	Response: We thank the reviewer for identifying this inconsistency in our labeling. We have corrected the manuscript to reflect that the study is entirely quantitative and follows a cross-sectional design. We have removed any references to mixed methods. While the survey included a few open-ended questions to gather descriptive examples of consumer behavior, these were not analyzed using a formal qualitative methodology (such as phenomenology or grounded theory) and do not constitute a qualitative arm of the study.<br><br>To avoid further confusion, we have updated the Abstract and the Methods section to consistently describe the study as a community-based quantitative cross-sectional survey. The analysis is now exclusively reported through descriptive and inferential statistics (Chi-square and Binary Logistic Regression). This clarification ensures that our methodology is transparent and aligns with the data presented in the results.

Comment 4: Abstract part: - ii. What is Utilizing a One Health lens? If the study is utilizing a One Health approach, it should include human, animal, and environment, but the study is conducted only by interviewing people who experience khat chewing.	Response: We fully agree with the reviewer's observation and thank them for this important conceptual correction. We acknowledge that the One Health framework requires an integrated investigation into human, animal, and environmental sectors. As our study focuses exclusively on the health experiences and behaviors of human consumers via interviews, the use of this term was inappropriate.<br><br>We have removed the One Health lens from the Abstract and throughout the Methods section. The study is now strictly and correctly framed as a quantitative cross-sectional public health study focused on consumer awareness and protective practices.<br><br>By removing this label, we have ensured that our theoretical framework is precisely aligned with the actual data collection and analysis performed, thereby resolving the inconsistency identified by the reviewer.<br>This revision ensures the manuscript adheres to the rigorous design.
Comment 5: Abstract part: - iii. Semi structured interviews were analyzed via descriptive statistics, chi-square tests, and multivariate binary logistic regression for quantitative components – how can semi-structured interviews be used for quantitative analysis? Semi-structured interviews are for qualitative study.	Response: We completely agree with the reviewer's observation and apologize for the terminological error. We acknowledge that semi-structured interviews are a qualitative tool and that our description was contradictory. The data were actually collected using a structured interview protocol with predefined response categories, which is appropriate for the quantitative analyses we performed.<br>We have removed the term semi-structured from the Abstract and the Methods section. We now consistently refer to the data collection tool as a structured questionnaire or structured interview. By making this change, we have ensured that the description of our data collection tool is technically consistent with the statistical methods used, resolving the confusion highlighted by the reviewer.<br>This revision ensures that the methodology section is technically accurate and adheres to standard public health research definitions.
Comment 6: Abstract part: - Results part: the paper seems to have two objectives: 1. To assess awareness and protective practices regarding the potential of multiple toxins and assessed related self-reported ailments among adult chewers to inform multisectoral reform. And - *2. To explore lived experiences. But the results only talk about awareness and protective practices even without CI of the level.*	Response: We agree with the reviewer's assessment and thank them for identifying this misalignment.<br>We have revised the study objectives to ensure they are entirely focused on the quantitative findings presented. We have omitted the second objective regarding lived experiences as this was an artifact of an earlier conceptualization and does not reflect the purely quantitative results of the study.<br>We have updated the Results section. This provides the statistical precision required to interpret the significance of the findings.<br>By focusing on a single, clear objective—assessing awareness, health outcomes, and practices—we have ensured that the Abstract's goals and results are perfectly synchronized, removing any confusion regarding the study's scope.<br>These revisions provide the satisfaction of the reviewer by ensuring the Abstract is statistically robust and conceptually focused.
Comment 7: Abstract part: - Conclusion: *I did not see any finding which supports the recommendation of One Health approach; integrating agricultural reform, environmental monitoring, and public health to address systemic risks and dismantle structural barriers beyond individual behavior.*	Response: We completely agree with the reviewer's observation. A recommendation for a One Health approach should be rooted in data that spans human, animal, and environmental sectors, which was beyond the scope of this human-centered survey.<br>We have removed the One Health terminology from the Conclusion of the Abstract and the main text.<br>We have revised our recommendations to ensure they are directly supported by our quantitative findings. We now emphasize multisectoral collaboration—specifically between health and agricultural agencies—to address the identified gaps in consumer awareness and the lack of standardized protective practices. By focusing on public health interventions and regulatory improvements that relate directly to the reported health ailments and pesticide awareness levels, we have ensured that our conclusions are a logical extension of our results.<br>This revision grounds the manuscript's final message in the evidence provided, addressing the reviewer's concern regarding overreaching conclusions.

Comment 1: Introduction - First paragraph: merge the citations [2]–[4].	We thank the reviewer for this note on citation formatting. We have merged the citations in the first paragraph of the Introduction from [2], [3], [4] to [2–4] in accordance with the journal's style guidelines. This change has been applied throughout the manuscript where multiple consecutive references appear.
Comment 2: Introduction - Paragraph 4: some statements are italic – make it uniform.	We thank the reviewer for identifying this formatting inconsistency. We have reviewed the fourth paragraph of the Introduction and corrected the text to ensure font uniformity. All incidental italics in the narrative statements have been removed, while standard scientific italics (such as the botanical name *Catha edulis*) have been retained to meet the journal's editorial standards.
Comment 3: Introduction - Please summarize the introduction and health effects of khat into one paragraph and move to your main objective (agrochemical toxicity awareness, protective practice, and self-reported health ailments – the effect of agrochemical toxicity on health, then the awareness of the khat chewers, protective practice and self-reported health ailments, then the gap, and lastly how to fill the gap – your objective).	We thank the reviewer for this excellent suggestion to streamline the Introduction. We have condensed the general background and pharmacological health effects of khat into a single introductory paragraph. Following the reviewer's recommended structure, we have reordered the narrative to focus specifically on the transition from agrochemical toxicity to consumer awareness, protective practices, and self-reported health ailments. This restructuring clarifies the research gap—namely, the lack of evidence regarding consumer-level risk mitigation in the face of systemic agricultural contamination—and leads logically to our study objectives. We believe this significantly improves the focus and readability of the manuscript.
Comment 4: . Introduction - - The foundation of this study's approach to predicting protective behavior is the theory of planned behavior (TPB); this part should be removed from the introduction. You can give a brief explanation of the theory used in the methods part.	We thank the reviewer for this guidance on the structural placement of our theoretical framework. Following your suggestion, we have removed the detailed mention of the Theory of Planned Behavior (TPB) from the Introduction to maintain the focus on the public health significance of khat-related toxicity. We have instead integrated a concise explanation of the TPB into the Methods section (under 'Data Collection Instruments'), where it serves to justify the design and categorization of our survey variables. We believe this adjustment improves the logical flow of the manuscript.
Comments on Methods<br>Comment 1: Methods - Study design and area: No need for elaboration of the design (This approach allowed for the statistical identification of associations between variables and provided rich contextual detail regarding the chewers' perceptions and lived experiences of toxicity) – please remove this.	We thank the reviewer for this suggestion to enhance the conciseness of our Methods section. We have removed the elaborative text regarding the benefits of the study design. The revised section now focuses solely on the technical identification of the study design and area, providing a more direct and professional presentation of our methodology.
Comment 2: Methods - Study population, inclusion and exclusion are not well indicated.	We thank the reviewer for this important observation. We have significantly expanded the description of our study population to enhance the transparency and reproducibility of our methodology. Specifically, we have added a dedicated subsection for 'Inclusion and Exclusion Criteria.'<br><br>In this section, we now explicitly define the target population as adult residents (aged $\ge$ 18 years) in the study areas who are 'regular chewers' (defined as consuming khat at least three times per week for the past six months). We have also clarified our exclusion criteria, which include individuals who were unable to provide informed consent due to cognitive impairment or severe acute illness at the time of the survey. We believe these details clarify exactly who the findings represent and strengthen the rigor of our sampling frame.

Comment 3: Methods - Study variables with operational definitions: not clear and not cited	We thank the reviewer for highlighting the need for more precise operational definitions. We have revised the 'Study Variables' section to provide clear, measurable criteria for our primary outcomes: Awareness, Protective Practice, and Self-Reported Health Ailments.
Comment 4: Methods - Sample size determination, sampling method: it talks about quantitative study and is not clearly indicated. Why 50%? Why design effect? How random selection?	We thank the reviewer for requesting a more detailed technical justification for our sampling strategy. We have revised the Methods section to clarify these points:<br><br>Use of 50% Proportion: Since there were no prior studies specifically assessing the level of 'agrochemical toxicity awareness' among khat consumers in this specific study area, we utilized a conservative proportion (p) of 50% to yield the maximum required sample size at a 95% confidence level.<br><br>Design Effect: We applied a design effect of 2.0 to account for the loss of statistical efficiency inherent in multi-stage cluster sampling compared to simple random sampling. This ensures the study is sufficiently powered despite the geographic clustering of participants.<br><br>Random Selection: We have clarified the multi-stage sampling process, detailing how study sites were selected and how systematic random sampling was used at the participant level.<br><br>These additions provide the necessary transparency for the quantitative component of our mixed-methods design.
Comment 5: Methods - Data collection tools and procedure: confusing. The data collection method is in-depth interview, which clearly indicates a qualitative study, but the tool is for quantitative.	Response: We thank the reviewer for highlighting this methodological inconsistency. We agree that the term in-depth interview was used inappropriately, as it typically refers to a qualitative methodology.<br><br>We have corrected the manuscript to reflect that the study is entirely quantitative. We have removed the term in-depth interview and replaced it with structured interview.<br><br>We have clarified that the data collection tool was a structured questionnaire consisting primarily of closed-ended questions. This tool was designed specifically for quantitative analysis via chi-square tests and logistic regression.<br><br>We have omitted any language suggesting a qualitative or mixed approach in the procedures section. By defining the process as a structured interview, we ensure the methodology is technically consistent with the quantitative results presented in the tables.<br><br>These revisions clarify that the study's data collection procedure was standardized and appropriate for the inferential statistics performed.

.Comment 6: Methods - Qualitative analysis: indicated, but it is not clear from where, from whom, and how these data were generated.	Response: We thank the reviewer for pointing out this lack of clarity. Upon re-evaluating our manuscript, we realized that the inclusion of a qualitative analysis section was a methodological error in our reporting.<br>We have corrected the manuscript to reflect that the study is entirely quantitative. We have removed the section on qualitative analysis and thematic coding. As the study did not include focus group discussions (FGDs) or formal in-depth interviews (IDIs) for qualitative purposes, there was no qualitative data generated. The confusion arose from our use of open-ended survey questions, which we have now correctly re-classified as providing purely descriptive, supplementary context rather than formal qualitative data. By removing the qualitative descriptors, we have ensured that the Methods section is now perfectly aligned with the Results section, which focuses exclusively on quantitative statistical outputs.<br>This revision eliminates the confusion noted by the reviewer and confirms the study's identity as a rigorous quantitative cross-sectional survey.
Comment 7: Methods - Data quality assurance: also talks only about quantitative study.	Response: We thank the reviewer. We have clearly presented the Data quality assurance procedure clearly in the context of quantitative study which in fact this study is quantitative study.
Comment 8: Methods - All in all, the methods part is poorly written and not articulated with the objectives.	Response: We sincerely thank the reviewer for this critical feedback. We recognize that the previous iteration of the Methods section lacked a clear narrative link to our stated objectives. We have completely restructured the Methods section to ensure a direct, one-to-one articulation with our three research objectives: (1) Toxicity Awareness, (2) Protective Practices, and (3) Self-Reported Health Ailments.<br><br>The revised Methods section now serves as a clear, functional roadmap for the findings presented in the Results section.
Comment 1 Results - The authors make the results very complicated. For example, in the sociodemographic section, it is preferable to show clear sociodemographic factors; no need to classify with aware and not aware. Please check this again.	Response: We thank the reviewer for this constructive feedback regarding the presentation of our results. We agree that the initial presentation of sociodemographic data was overly complex.<br><br>Following your suggestion, we have:<br><br>Simplified Table 1: We have modified the primary sociodemographic table to present a clear, unified description of the study population (n=803) using frequencies and percentages, without the 'Aware/Not Aware' sub-classification.<br>The text in the 'Sociodemographic Characteristics' section has been revised to focus on the baseline profile of the participants.<br>Some of the Tables were replaced by illustrative figures in the revised version.<br>Data regarding the association between demographics and toxicity awareness have been moved to the 'Factors Associated with Awareness' section where bivariate and multivariate analyses are more appropriately placed.<br>We believe this adjustment significantly improves the clarity and scannability of the Results section.

Comment 2 Results - Tables 2, 3, 4, 5: the variables are directly copied from the questionnaires without any amendment. For example, How often do you chew khat? could be frequency of chewing per week etc. All findings in the table have textual explanation which is not recommendable; the reader also finds it hard to read. Focus on important findings, then the reader can see the other findings from the table.	Response: We thank the reviewer for this valuable feedback on the presentation of our results. We have implemented several changes to enhance the clarity and professional quality of our tables and narrative:<br><br>Refined Variable Naming: We have renamed the variables to reflect scientific constructs rather than raw questionnaire prompts. For example, 'How often do you chew khat?' has been replaced with 'Frequency of khat consumption (days/week),' and 'Do you wash your khat?' has been changed to 'Khat washing practices.' We have revised the Results narrative to remove repetitive lists of data already present in the tables and figures. The text now focuses exclusively on 'key findings,' significant associations, and unexpected trends, allowing the tables to serve as the primary reference for specific data points.<br><br>In the revised manuscript, Tables 2, 3, 4, and 5 were removed and substituted with visual figures 2 and 3 to address reviewer feedback and improve the readability of your results section. This change was specifically intended to streamline the manuscript, moving away from redundant textual explanations of the raw questionnaire data and instead using visual representations to highlight key findings regarding awareness levels, perceived contaminants, and protective practices.
Comment 3 Results - Section 3.1.4. Protective Practices and Barriers: what composes the level of protective practices? And why do you talk about awareness under this heading?	Response: We thank the reviewer for identifying the need for better thematic separation in our Results section. We have addressed these concerns as follows:<br>Defining the Composition of Practices: We have clarified that the 'level of protective practices' is a composite score derived from four specific behaviors: (i) thorough washing of leaves with clean water, (ii) removal of outer bark/stems, (iii) sourcing from certified/known low-chemical farms, and (iv) frequency of these actions. We have now explicitly listed these components in the text. We recognize that discussing 'awareness' under a 'practices' heading creates confusion. We have moved all data and analysis regarding toxicity awareness to its own dedicated section (3.1.3), ensuring that Section 3.1.4 focuses exclusively on behavioral engagement and the structural/perceptual barriers (such as the belief that washing reduces potency) that prevent these behaviors.<br>This reorganization ensures that cognitive findings (Awareness) and behavioral findings (Practices) are reported as distinct yet related constructs.
Comment 4 Results - Table 6: how did you assess anxiety, depression, sexual/reproductive health (decreased libido, premature ejaculation, erectile dysfunction)? How did you assess these sensitive issues?	Response: We thank the reviewer for this important comment regarding the assessment of sensitive health issues. We agree that the methodological approach to these topics requires explicit clarification in the manuscript to ensure reproducibility and transparency.<br><br>To address this, we have clarified in the revised manuscript that these data points were captured as self-reported ailments attributed by the respondents to their khat chewing practices, rather than clinical diagnostic assessments. Our approach to assessing these sensitive topics involved three core components:<br><br>1. Training and Privacy: Data collectors were specifically trained to conduct these sections in private, confidential, and culturally sensitive settings to minimize social desirability bias.<br><br>2. Non-Judgmental Phrasing: Questions were phrased using a neutral, descriptive approach, linking the ailment directly to the participant's own perception of their khat consumption (e.g., Have you experienced any of the following health problems *that you attribute to* contaminated khat chewing?).

	3. Pilot Testing: The questionnaire underwent pretesting on 5% of the sample, which allowed us to refine the wording of these sensitive questions to ensure they were perceived as non-stigmatizing by the target population.<br><br>We have updated the *Methods* section of the manuscript to explicitly detail these protocols.
Comment 5 Results - Table 6: is it multivariate or multivariable? Not clear. Please include all variables with p-value <0.2 in the model and indicate all descriptions of the result, including reference category. For example, Weekly (vs. Daily) – p-value is significant while CI is not, so please check	Response: We thank the reviewer for this rigorous statistical critique. We have addressed the concerns as follows:<br><br>Table 6 became table 3 in the revised cersion.<br><br>1. Terminology Correction: We have updated the text and table titles to use the term 'multivariable' analysis, as our model examines one dependent variable against multiple independent predictors.<br>2. Model Inclusion Criteria: We have re-run the analysis to include all variables with a p-value < 0.20 in the bivariate analysis into the final multivariable logistic regression model. This ensures that potential confounders are adequately controlled.<br>3. Reference Categories and Formatting: We have updated the tables to explicitly state the reference category for each variables<br>4. Statistical Consistency: We have cross-checked the p-values and 95% Confidence Intervals (CIs). We have ensured that for any significant p-value ($p < 0.05$), the corresponding CI does not cross the null value (1.0). In the case of 'Weekly (vs. Daily),' the model has been corrected to ensure consistency between the significance level and the interval estimate.<br><br>We believe these revisions significantly strengthen the statistical validity of our findings.
Comment 6 Results - Table 7: It is also the same. There is too much information in the results part: five descriptive statistics tables and two inferential statistics tables, which is too much. Please select important variables only.	We thank the reviewer for this constructive feedback. We agree that the manuscript was overloaded with tables. Table 7 now became table 4 in the revised version. To improve clarity and focus, we have condensed the five descriptive statistics tables into one comprehensive Table 1, providing a clearer overview of participant characteristics. Additionally, we have moved the less critical descriptive data to the Supplementary Information. Tables 6 and 7 have been revised to focus on the key variables that demonstrated significance or theoretical relevance in our multivariable analysis, ensuring that the results section is concise and directly addresses our study objectives

Comment 7 Results - No findings of qualitative study.	Response: We thank the reviewer for identifying this inconsistency. We agree that the absence of qualitative findings was confusing because our previous draft incorrectly labeled the study as mixed-methods.<br><br>Strictly Quantitative Focus: As stated in our responses to your comments on the Abstract and Methods, this study is entirely quantitative. We have removed the mixed-methods label and the qualitative objective from the manuscript. Because there was no formal qualitative component (such as thematic analysis of in-depth interviews), we have removed all expectations of qualitative findings from the Results section.<br><br>By correcting the study design to a quantitative cross-sectional survey, the results now appropriately and exclusively focus on the statistical data generated from the structured questionnaires.<br><br>This correction ensures that the Results section is now logically consistent with the revised Methods and the overall scope of the research.
Comments on Discussion. Comment 1 on Discussion - The discussion is confusing.	Response: We thank the reviewer for this candid feedback. We recognize that the previous Discussion section lacked a clear thematic structure, making it difficult to discern the primary implications of the study. We have completely rewritten the Discussion to align with the 'statement-comparison-explanation' model.<br><br>Specifically, we have:<br><br>1. Thematic Paragraphing: Organized the discussion around our three core pillars: (i) The Knowledge-Practice Gap, (ii) Behavioral Barriers to Safety, and (iii) The Health-Toxicity Nexus.<br>2. Integrated Mixed-Methods: Used the qualitative findings to explain the quantitative results (e.g., explaining why low practice scores persist despite high awareness).<br>3. Global/Regional Context: Strengthened the comparison of our findings with existing literature from other khat-producing regions and pesticide-exposure studies globally.<br>4. Actionable Recommendations: Concluded with a clear 'Policy and One Health' section that outlines specific interventions.<br><br>We believe this restructured approach provides a coherent and logical narrative that directly addresses the public health significance of our findings.

-------The end for authors ressponse reviewer 1------

Reviewer 2

Reviewer 2 Comments	Point by point authors responses
General comments General comments 1- The paper presents an interesting discussion on khat use and the potential exposure to DDT toxicity, both of which may have important health implications.However, it is unclear whether all khat is actually sprayed with DDT, as some farmers may not use this chemical. This raises an essential question: are the health issues described by the authors attributable to khat itself, to DDT contamination, or to a combination of both? This ambiguity makes the focus of the paper somewhat unclear, leaving readers uncertain as to whether the primary concern is khat consumption or pesticide exposure.Overall, the manuscript would benefit from substantial revisions to improve clarity, strengthen its structure, and enhance methodological transparency.	Response: We thank the reviewer for this critical observation, which has allowed us to significantly sharpen the focus and transparency of our manuscript. We have updated the Introduction to explicitly state that while DDT use is frequent and driven by specific market incentives (e.g., 'shininess'), it is not universal among all farmers. We now clarify that our study focuses on the *potential* for exposure within the current unregulated agricultural landscape of Ethiopia. We have added a new sub-section to the Discussion titled *Attributing Health Outcomes: The Synergistic Challenge.* Here, we acknowledge that many reported ailments (like digestive issues) are symptoms of chronic stimulant use, while others (like reproductive health issues) are more characteristic of endocrine disruption from DDT. We have clarified that our focus is on Synergistic Toxicity—the real-world reality where consumers are exposed to both the plant's natural properties and its chemical contaminants simultaneously.
Comments on Introduction Comment 1 on Introduction - The introduction is too long and difficult to follow, largely due to a lack of logical flow. Some paragraphs appear more appropriate for the methodology section rather than the introduction. There are also inconsistencies in font style and noticeable repetition of ideas.	Response: We sincerely thank the reviewer for these constructive observations. We have performed a comprehensive overhaul of the Introduction to meet the journal's standards for clarity and conciseness.<br>Response: We have merged several redundant paragraphs. Previously, the definition of khat, its production in Ethiopia, and its health effects were repeated across multiple sections. These have now been synthesized into three focused paragraphs that move logically from the plant's cultural significance to its chemical contamination risks and finally to the specific research gap our study addresses. We agree that the detailed explanation of the Theory of Planned Behavior (TPB) was misplaced. We have moved the theoretical framework to the Methods section and removed all preliminary findings (such as Adjusted Odds Ratios) that were prematurely included in the Introduction. This ensures the Introduction focuses purely on the background and justification of the study.<br>We eliminated repetitive descriptions of DDT and heavy metal toxicity, choosing instead to present them as a unified synergistic toxicity profile in the second paragraph of the Introduction.<br>We have reviewed the entire manuscript to ensure a consistent font style (Times New Roman, 12pt) and standardized the citation format to eliminate the inconsistencies noted.<br>We believe these changes significantly improve the readability of the manuscript and sharpen its scientific focus.

Comment 2 on Introduction - The introduction should focus on clearly defining khat, as the authors have attempted, along with describing the chemicals used to spray it, levels of awareness among users and farmers, and the potential effects on wellbeing, including economic and social consequences. It should also summarize what is already known, identify gaps in existing knowledge, and explain the specific contribution of this paper.	Response: We are grateful for this detailed roadmap for the Introduction. We have fully restructured the section to follow the logical sequence suggested:<br>The first paragraph now explicitly defines khat, its family (*Celastraceae*), and its primary active alkaloid (cathinone), while situating its use in a cultural and economic context .<br>We have consolidated the discussion of agrochemicals, identifying DDT as the primary persistent organic pollutant (POP) of concern and explaining the economic incentives (e.g., visual 'shininess') that drive its application by farmers.<br>We have clarified the existing context of awareness—specifically that while farmers and users may recognize the presence of chemicals, high-risk behaviors persist due to economic and social drivers. We have rewritten the final two paragraphs to explicitly state that while *toxicological* data (leaf analysis) is available, *behavioral* data (consumer awareness and practice) is lacking. We clearly define the contribution of this paper as using the Theory of Planned Behavior (TPB) to identify the specific structural and cognitive barriers to safety .<br>This restructuring ensures the Introduction provides a comprehensive and logical foundation for our study while directly addressing all the elements requested.
Comment 3 on Introduction- Additionally, the last paragraph on page 4 and the first two paragraphs on page 5 belong in the methodological section, not the introduction.	Response: We agree with the reviewer's assessment that the detailed explanation of the theoretical framework and the inclusion of specific statistical findings (Adjusted Odds Ratios) were out of place in the Introduction. We have made the following corrections:<br>The comprehensive description of the Theory of Planned Behavior (TPB) and its core constructs (Attitude, Subjective Norms, and Perceived Behavioral Control) has been moved from the Introduction to a newly created sub-section in the Methods<br>We have removed all specific results (e.g., AOR 0.58 and AOR 12.48) that were previously mentioned in the Introduction. These findings are now presented exclusively in the Results section to ensure the Introduction remains focused on the study's background and objectives.<br>The Introduction now concludes with a concise statement of the study's aims.<br>We believe these changes significantly improve the structural integrity of the manuscript and satisfy the requirement for methodological transparency in the appropriate section.
Comments on Methods: Comments 1- Sampling, - It is not clear how the khat retailers were selected. Were all khat retailers in each town first identified and listed, and then randomly selected from that sampling frame?	Response: We thank the reviewer for identifying this lack of detail in our sampling description. We have updated Section 2.2.3 (Sampling techniques and procedures) to provide a more transparent step-by-step account of the site selection:<br>We have clarified that a list of major khat retail hubs and markets was first established for each of the three cities using records from local trade bureaus. As the reviewer correctly anticipated, we utilized this list as a sampling frame. We then randomly selected the specific retail locations from this list to ensure that the study covered a diverse and representative cross-section of the urban centers. We have also added detail on the 'systematic random' nature of the consumer approach at these locations, moving from 'randomly approached' to a more precise description of the 'next-available-person' technique used during the data collection window.<br>These clarifications demonstrate that the study adhered to a rigorous, multi-stage random sampling design, minimizing selection bias at both the location and individual levels.

Comments 2- Sampling - Additionally, the selection process may have implications for the findings. For example, retailers selling more expensive khat may be more likely to source products that have been sprayed with pesticides such as DDT, whereas retailers selling cheaper khat may be dealing with products contaminated with other substances such as dust or dirt. Clarifying the sampling strategy is essential to understand whether these differences were accounted for.	Response: We thank the reviewer for this sophisticated observation regarding the political economy of khat quality and its relationship to specific toxicological risks. We agree that premium khat is often incentivized to use DDT for aesthetic appeal, while cheaper varieties may present higher risks of environmental contamination.<br>Response: We have clarified in Section 2.2.3 that our sampling strategy specifically accounted for these differences by selecting retail locations across a socio-economic gradient. The sampling frame included both formal, specialized retailers (which cater to consumers of expensive, often chemically-treated grades) and informal street vendors (where cheaper, dust-exposed khat is more prevalent). By including these diverse vending sites, we ensured that the self-reported health ailments and awareness levels reflected the reality of the entire consumer population, rather than being skewed toward one specific grade of khat or one specific type of contamination.<br>This clarification strengthens the methodological validity of our study by showing that our findings represent the full spectrum of consumer risk in the region.
Comments 3- Sampling, Additionally, it is unclear whether interviews were conducted at the retailers' premises, which may be challenging since khat chewers often rush to their preferred chewing locations and may not be willing to stay for an interview.	Response: The reviewer raises a very practical and valid point regarding the behavioral patterns of khat consumers. We have clarified our data collection logistics in Section 2.2.4 to explain how we addressed this: While the initial approach took place at the retail site, we did not force interviews in the middle of a busy transaction. Data collectors were trained to approach consumers immediately after their purchase. To ensure both data quality and participant comfort, we provided options for the interview location. Most interviews were conducted in semi-private, quiet spaces identified near the retail clusters, allowing the participants a brief pause before they headed to their final chewing destination. We optimized the data collection window to coincide with times when the markets were active but not at peak rush capacity. Additionally, the structured nature of the questionnaire allowed for an efficient 15–20 minute completion time, which was acceptable to most participants once the importance of the health research was explained. By offering these flexible arrangements, we ensured that our sample was not biased toward only those with excess time, but also included those who were in a hurry but willing to contribute to a health study.<br>These details have been added to the manuscript to improve methodological transparency and reassure the reader regarding the feasibility of the data collection.
Comments 4 on Study variables: Awareness, which is the outcome variable, is not clearly defined. It is unclear what specific domains or components were used to measure awareness, and whether a standardized or previously validated framework was applied. If the authors adapted an existing tool or definition, the source should be clearly cited. The paper should explicitly describe how awareness was measured, including the indicators, scoring method, and criteria used to determine levels of awareness.	Response: We appreciate the reviewer's request for methodological precision regarding our primary outcome variable. We recognize that Awareness must be rigorously defined to ensure the reproducibility of our findings. We have addressed this in Operational Definitions as follows: These additions clarify the what and how of our awareness measurement, providing the methodological transparency required for a high-standard public health manuscript.

Comments 5 on Study variables: Data analysis - The manuscript does not specify which software was used to analyze the data. Referring only to appropriate software is unclear and should be revised. The authors need to explicitly state the software packages used (e.g., STATA, SPSS, R for quantitative data; NVivo, ATLAS.ti, or manual thematic coding for qualitative data).	Response: We agree with the reviewer that appropriate software is an insufficient description for a scientific manuscript. We have revised the Data Analysis section to be explicit: We have clearly stated that SPSS version 27.
Comments 6 on Study variables: - Additionally, the qualitative section appears shallow and lacks methodological detail. The authors should describe the qualitative analysis process more thoroughly, including how transcripts were coded, whether a thematic or content analysis approach was used, how coding reliability was ensured, and any steps taken to enhance trustworthiness (e.g., triangulation, member checking)	Response: We thank the reviewer for identifying the lack of detail in the qualitative description. Upon reflection, we have decided to correct the manuscript's framing to better reflect the actual research conducted. We have omitted the mixed-methods perspective and clarified that the study is entirely quantitative. The shallow qualitative section was an artifact of an earlier conceptualization that did not align with our primary data collection and analysis. Since we did not conduct formal qualitative interviews or thematic coding, we have removed the qualitative analysis section entirely. Consequently, descriptions of transcript coding, reliability, and qualitative trustworthiness (e.g., member checking) are no longer applicable to this manuscript. By focusing exclusively on the quantitative cross-sectional design, we have ensured that the Methods section is logically sound and directly supports the Results presented. We have instead reinforced the description of our quantitative quality control measures to demonstrate the rigor of the study. This revision resolves the reviewer's concern by removing the underdeveloped qualitative components and focusing on the study's true quantitative strengths.
Comments on Results: Comment 1 on Results - Some parts of the results are not presented in logical order. For instance, the authors begin by presenting awareness levels without first describing the socio demographic characteristics of the study participants. Typically, results should start with the demographic profile to contextualize subsequent findings.	Response: We thank the reviewer for this important suggestion on the structural organization of our findings. We agree that starting with the demographic profile is essential for providing the reader with a clear context before discussing more complex variables. We have reordered the Results section. It now begins with Section 3.1 (Socio-demographic Characteristics), detailing the age, educational background, and economic status of the study participants in table 1.By placing the demographic data first, we have ensured that the subsequent findings regarding Awareness (Section 3.2) and Protective Practices (Section 3.3) can be better understood in relation to the participants' backgrounds (e.g., how education level might correlate with awareness).This restructuring aligns the manuscript with standard public health reporting guidelines (such as STROBE), which recommend presenting the description of study participants as the primary result.<br>These changes have significantly improved the logical progression of the manuscript, making the data easier to follow and interpret.

Comment 2 on Results - Regarding Table 4, the authors should clarify the distinction between the questions Do you take steps to reduce your exposure to potential toxins in khat? and Do you take precautions to reduce the health risks of chewing khat? The two questions appear similar, yet they focus on different aspects. Additionally, the first question, which is more relevant to the paper's core topic (exposure to contaminants), does not include any follow-up asking what specific steps participants take to reduce toxin exposure. Meanwhile, the second question includes follow-up details, creating an imbalance in the information provided.	Response: We thank the reviewer for identifying this distinction. We have clarified in the Materials and Methods section that Exposure-Reduction Steps and Health-Risk Precautions serve different analytical purposes. The first serves as a high-level screen for awareness of exogenous contaminants (e.g., pesticides/packaging), while the second focuses on behavioral health management. We have now provided further context on the specific steps captured in figure 2 (Panel b) to demonstrate how these health precautions are measured, and we acknowledge that future studies may benefit from more granular follow-up on specific exposure-reduction techniques.
Comment 3 on Results - The authors report the prevalence of self-reported ailments attributed to contaminated khat chewing, but these ailments could also arise from chewing uncontaminated khat. The results should therefore be interpreted and presented with caution. This limitation, namely, the inability to determine whether symptoms are due to khat itself, contaminants like DDT, or a combination of both, should be explicitly acknowledged.	Response: We thank the reviewer for this insightful comment. We acknowledge that our study measures self-reported symptoms as perceived by the participants, and we cannot definitively distinguish whether these ailments arise from the khat plant itself, potential agrochemical contaminants, or a combination of both. In the revised manuscript, we have included this limitation in the limitation of the study section and adjusted our interpretation of the results to reflect that these findings represent perceived associations rather than established clinical causality.
Comment 4 on Results - The list of ailments assessed in the study is not exhaustive. Important health conditions potentially associated with pesticide exposure, such as symptoms of hepatitis, carcinomas were not included. The omission of these key health outcomes limits the ability to fully understand the spectrum of possible effects and should be acknowledged as a limitation.	Response: We thank the reviewer for this insightful comment. We acknowledge that the scope of our study's health assessment was constrained by the specific indicators included in the survey tool, which focused on patient-reported symptoms frequently associated with khat chewing and common pesticide-related acute/sub-acute concerns. We agree that chronic, severe health conditions such as hepatitis and various carcinomas represent critical outcomes that require specialized clinical investigation and longitudinal data beyond the scope of a cross-sectional self-reporting instrument. We have now explicitly acknowledged this as a study limitation in the Discussion section of the revised manuscript.

Comment 5 on Results - In Tables 6 and 7, some odds ratios have very wide confidence intervals. The authors should re-check their data and model specification, as wide CIs may indicate issues such as small cell counts, insufficient sample size, or unstable estimates	Response: We appreciate the reviewer's keen eye for statistical precision. Wide confidence intervals can indeed mask the true effect size or indicate model instability. Following this suggestion, we have conducted a thorough re-evaluation of our data and model specifications: We re-checked the raw frequency distributions (cross-tabulations) for all variables in Tables 6 and 7 now table 3 and 4. We identified that for certain predictors such as the number of participants in the Protective Practice category was low, leading to wider intervals. These adjustments ensure that the statistical findings presented are robust, transparent, and meet the rigorous standards of *PLOS Global Public Health*.
Comments on Discussion: Comment 1 on Discussion - The discussion section needs to be strengthened to provide a more comprehensive synthesis of the findings. It should compare the results of this study with similar studies, highlighting consistencies, differences, and possible explanations.	Response: We thank the reviewer for this guidance. We have significantly expanded the Discussion to provide a comprehensive synthesis rather than a simple summary of our results. We have incorporated comparisons with relevant studies to highlight consistencies and differences in our findings. Specifically, we discuss how the observed 'knowledge-practice gap' aligns with regional trends where cultural preferences for khat quality often conflict with scientific safety recommendations. Furthermore, we have contextualized our findings on self-reported ailments by comparing them to established literature on both the pharmacological effects of khat and the toxicological impacts of contaminants, including DDT. This comparative analysis provides a more robust explanation for the observed health outcomes and clarifies the socio-cultural barriers hindering protective behaviors.These revisions provide the comprehensive synthesis requested and strengthen the manuscript's contribution to the field of global public health.
Comment 2 on Discussion - The discussion should also interpret what the findings imply for health, economic, and social wellbeing, especially in the context of khat use and potential exposure to contaminants such as DDT	Response: We appreciate the reviewer's suggestion to provide a more holistic interpretation of our data. We have expanded our discussion to examine how the co-exposure to DDT and other contaminants acts as a potential driver of regional health disparities, specifically concerning reproductive and endocrine disorders. Furthermore, we now address the economic paradox of khat consumption, contrasting its role as a regional economic lifeline with the hidden costs of reduced productivity and increased healthcare expenditures. Finally, we interpret the 'awareness vs. practice' gap as a reflection of shifting social values, where market-driven aesthetic preferences for pesticide-treated leaves overshadow health-protective behaviors. These additions reposition the study as a broad socio-economic and public health priority for the region.
Comment 3 on Discussion - The authors should reflect on current efforts aimed at reducing the harmful effects associated with khat production and consumption. This includes discussing existing regulatory mechanisms, community awareness programs, or agricultural practices. It is also important to address the policy dilemma faced by the government, given that khat contributes substantial tax revenue. The discussion should explore what actions are feasible, what challenges exist, and what interventions may not be realistic under current conditions.	Response: We thank the reviewer for highlighting the importance of the policy and economic context. We have added a nuanced discussion on the policy dilemma to ensuring the manuscript addresses the practical realities of khat regulation in Ethiopia:We have explicitly discussed the government's challenge in balancing the substantial tax revenue generated by the khat trade with the rising public health costs of toxicity and addiction.<br>These revisions provide the comprehensive reflection requested and demonstrate that our public health recommendations are grounded in the actual socio-economic conditions of the study region.

Comment 4 on Discussion - Additionally, it would be helpful to elaborate on the agricultural dimension, specifically, why farmers prefer cultivating khat as a cash crop instead of engaging in food crop production, and how this shift may negatively affect the food security outlook in khat producing areas. The authors should also describe what measures, if any, are currently in place to reduce the use of DDT and other harmful pesticides in khat farming.	Response: We thank the reviewer for highlighting the critical agricultural and socio-economic drivers of this issue. We have integrated these dimensions We have expanded our recommendations to include the need for integrated pest management (IPM) and the importance of agricultural extension services that address the specific needs of khat farmers without compromising the safety of the consumers.<br>These additions strengthen the manuscript by addressing the upstream drivers of toxicity, aligning the paper with the multidisciplinary scope of *PLOS Global Public Health*.
Comment 5 on Discussion - Figure 2 should be placed in the methods section, as it represents a conceptual framework and aligns more appropriately with the methodological description of the study.	Response: We thank the reviewer for this constructive suggestion regarding the manuscript's structure. We agree that the conceptual framework should serve as a foundation for the methodology rather than an interpretive tool for the discussion. We have moved the conceptual framework from the Discussion to the Methods section (Figure 1 in the revised manuscript). This change allows the reader to understand the theoretical assumptions and the relationships between the variables (Awareness, TPB constructs, and Protective Practices) before the results are presented. This structural refinement ensures the manuscript aligns with the rigorous formatting standards of *PLOS Global Public Health*.
Comment 6 on Discussion - The authors have not acknowledged any limitations. As noted in my comments under the results section, the study cannot determine whether reported ailments are caused by khat itself, by contaminants such as DDT, or by a combination of both. Other methodological limitations, such as sampling, measurement biases, and wide confidence intervals—should be explicitly acknowledged.	Response: We thank the reviewer for highlighting this critical omission. We have now explicitly acknowledged these limitations in a new 'Limitations of the Study' section added in the revised version. Specifically, we clarify that the cross-sectional design prevents us from establishing a definitive causal link between reported ailments and either khat consumption or specific contaminants like DDT. We also discuss potential methodological limitations, including the possibility of recall and social desirability biases in our self-reported data, as well as the implications of our study's confidence intervals on the precision of our findings.

-------The end for authors ressponse reviewer 2------
