# Supplementary material for: Khat consumers exposure to toxicity: Agrochemical toxicity awareness, protective practice and self-reported health ailments among khat consumers in Ethiopia
Source: PLOS Glob Public Health. 2026 Jul 21;6(7):e0006913. doi: 10.1371/journal.pgph.0006913 (PMC13387539; doi:10.1371/journal.pgph.0006913)
Supplement: Attachment — Submitted filename: Response to Reviewers .pdf [file pgph.0006913.s006.pdf]

Point by point responses

Khat Consumers Exposure to Toxicity: Agrochemical Toxicity Awareness, Protective Practice and Self-Reported Health Ailments Among Khat Consumers in Ethiopia-PLOS Global Public Health - PGPH-D-26-00831R1

Subject: Resubmission of Manuscript [PGPH-D-26-00831R1**]**

Dear Editor,

Thank you for the opportunity to revise our manuscript. We have carefully addressed all of your comments and verified that the manuscript meets the journal's technical requirements. Below, we provide a point-by-point summary of the actions taken:

**Comment/Requirement**	**Author Response**
**Reference Style:** Correct citation format throughout the manuscript (e.g., [1, 5-7] instead of [1], [5]–[7]).	We thank the editor for this observation. We have carefully revised the manuscript to ensure that all in-text citations strictly adhere to the PLOS citation style. We have updated our reference management settings to the standard PLOS format, which ensures that multiple citations are now correctly grouped within a single set of square brackets (e.g., [1, 5–7]) throughout the entire text. All citations have been reformatted accordingly, and the reference list has been verified for consistency with the journal's guidelines.
**Embedded Figures:** Remove all figures embedded in the main manuscript text.	**Response:** Thank you. We have removed all figures from the main manuscript text in accordance with the journal's guidelines. We have replaced each embedded image with a clear placeholder ([Insert Figure 1 here]<br>[Insert Figure 2 here]<br>[Insert Figure 3 here]<br>to indicate the intended placement. All figures have been uploaded as separate, high-resolution files in the submission system as requested.
**Figure Files:** Submit each figure as a separate high-resolution file (.tif or .eps) under 10MB each.	**Response:** Thank you. We have prepared all figures in accordance with the journal's technical specifications. Each figure has been exported as a separate high-resolution file in the requested format (.tif) and is under

	10MB. We have uploaded these files individually to the submission system, ensuring they meet the required quality standards for publication.
**PLOS NAAS Tool:** Verify figure files using the PLOS NAAS tool.	**Response:** Thank you. We have processed all figure files through the PLOS NAAS tool to ensure they meet all technical requirements and standards for publication.
**Supporting Information Legends:** Include a full list of legends for supporting information files before or after the reference list.	**Response:** Thank you. A comprehensive list of legends for all supporting information files has been added immediately following the reference list.
**Reference Verification:** Review reference list to ensure there are no retracted publications.	**Response:** Thank you. We have conducted a thorough audit of all cited literature. We confirm that there are no retracted publications in our reference list; all cited sources are current and valid.

We believe these revisions fully satisfy the journal's requirements and further improve the quality of our submission. Thank you for your continued time and guidance in evaluating our work.

Sincerely,

The Corresponding author
