# Supplementary material for: Khat consumers exposure to toxicity: Agrochemical toxicity awareness, protective practice and self-reported health ailments among khat consumers in Ethiopia
Source: PLOS Glob Public Health. 2026 Jul 21;6(7):e0006913. doi: 10.1371/journal.pgph.0006913 (PMC13387539; doi:10.1371/journal.pgph.0006913)
Supplement: Attachment — Submitted filename: Comments PGPH.docx [file pgph.0006913.s004.docx]

Comments PGPH

**General comments**

The paper presents an interesting discussion on khat use and the potential exposure to DDT toxicity, both of which may have important health implications. However, it is unclear whether all khat is actually sprayed with DDT, as some farmers may not use this chemical. This raises an essential question: are the health issues described by the authors attributable to khat itself, to DDT contamination, or to a combination of both? This ambiguity makes the focus of the paper somewhat unclear, leaving readers uncertain as to whether the primary concern is khat consumption or pesticide exposure. Overall, the manuscript would benefit from substantial revisions to improve clarity, strengthen its structure, and enhance methodological transparency.

**Introduction**

The introduction is too long and difficult to follow, largely due to a lack of logical flow. Some paragraphs appear more appropriate for the methodology section rather than the introduction. There are also inconsistencies in font style and noticeable repetition of ideas.

The introduction should focus on clearly defining khat, as the authors have attempted, along with describing the chemicals used to spray it, levels of awareness among users and farmers, and the potential effects on wellbeing, including economic and social consequences. It should also summarize what is already known, identify gaps in existing knowledge, and explain the specific contribution of this paper.

Additionally, the last paragraph on page 4 and the first two paragraphs on page 5 belong in the methodological section, not the introduction.

**Methods**

**Sampling**

It is not clear how the khat retailers were selected. Were all khat retailers in each town first identified and listed, and then randomly selected from that sampling frame? Additionally, the selection process may have implications for the findings. For example, retailers selling more expensive khat may be more likely to source products that have been sprayed with pesticides such as DDT, whereas retailers selling cheaper khat may be dealing with products contaminated with other substances such as dust or dirt. Clarifying the sampling strategy is essential to understand whether these differences were accounted for. Additionally, it is unclear whether interviews were conducted at the retailers' premises, which may be challenging since khat chewers often rush to their preferred chewing locations and may not be willing to stay for an interview.

**Study variables**

Awareness, which is the outcome variable, is not clearly defined. It is unclear what specific domains or components were used to measure awareness, and whether a standardized or previously validated framework was applied. If the authors adapted an existing tool or definition, the source should be clearly cited. The paper should explicitly describe how awareness was measured, including the indicators, scoring method, and criteria used to determine levels of awareness.

**Data analysis**

The manuscript does not specify which software was used to analyze the quantitative and qualitative data. Referring only to “appropriate software” is unclear and should be revised. The authors need to explicitly state the software packages used (e.g., STATA, SPSS, R for quantitative data; NVivo, ATLAS.ti, or manual thematic coding for qualitative data).

Additionally, the qualitative section appears shallow and lacks methodological detail. The authors should describe the qualitative analysis process more thoroughly, including how transcripts were coded, whether a thematic or content analysis approach was used, how coding reliability was ensured, and any steps taken to enhance trustworthiness (e.g., triangulation, member checking).

**Result**

Some parts of the results are not presented in logical order. For instance, the authors begin by presenting awareness levels without first describing the socio‑demographic characteristics of the study participants. Typically, results should start with the demographic profile to contextualize subsequent findings.

Regarding Table 4, the authors should clarify the distinction between the questions “Do you take steps to reduce your exposure to potential toxins in khat?” and “Do you take precautions to reduce the health risks of chewing khat?” The two questions appear similar, yet they focus on different aspects. Additionally, the first question, which is more relevant to the paper’s core topic (exposure to contaminants), does not include any follow-up asking what specific steps participants take to reduce toxin exposure. Meanwhile, the second question includes follow-up details, creating an imbalance in the information provided.

The authors report the Prevalence of Self‑Reported Ailments Attributed to Contaminated Khat Chewing, but these ailments could also arise from chewing uncontaminated khat. The results should therefore be interpreted and presented with caution. This limitation, namely, the inability to determine whether symptoms are due to khat itself, contaminants like DDT, or a combination of both, should be explicitly acknowledged.

The list of ailments assessed in the study is not exhaustive. Important health conditions potentially associated with pesticide exposure, such as symptoms of hepatitis, carcinomas were not included. The omission of these key health outcomes limits the ability to fully understand the spectrum of possible effects and should be acknowledged as a limitation.

In Tables 6 and 7, some odds ratios have very wide confidence intervals. The authors should re-check their data and model specification, as wide CIs may indicate issues such as small cell counts, insufficient sample size, or unstable estimates.

**Discussion**

The discussion section needs to be strengthened to provide a more comprehensive synthesis of the findings. It should compare the results of this study with similar studies, highlighting consistencies, differences, and possible explanations. The discussion should also interpret what the findings imply for health, economic, and social wellbeing, especially in the context of khat use and potential exposure to contaminants such as DDT.

The authors should reflect on current efforts aimed at reducing the harmful effects associated with khat production and consumption. This includes discussing existing regulatory mechanisms, community awareness programs, or agricultural practices. It is also important to address the policy dilemma faced by the government, given that khat contributes substantial tax revenue. The discussion should explore what actions are feasible, what challenges exist, and what interventions may not be realistic under current conditions.

Additionally, it would be helpful to elaborate on the agricultural dimension, specifically, why farmers prefer cultivating khat as a cash crop instead of engaging in food crop production, and how this shift may negatively affect the food security outlook in khat‑producing areas. The authors should also describe what measures, if any, are currently in place to reduce the use of DDT and other harmful pesticides in khat farming.

Figure 2 should be placed in the methods section, as it represents a conceptual framework and aligns more appropriately with the methodological description of the study.

The authors have not acknowledged any limitations. As noted in my comments under the results section, the study cannot determine whether reported ailments are caused by khat itself, by contaminants such as DDT, or by a combination of both. Other methodological limitations, such as sampling, measurement biases, and wide confidence intervals—should be explicitly acknowledged.
