# Supplementary material for: Khat consumers exposure to toxicity: Agrochemical toxicity awareness, protective practice and self-reported health ailments among khat consumers in Ethiopia
Source: PLOS Glob Public Health. 2026 Jul 21;6(7):e0006913. doi: 10.1371/journal.pgph.0006913 (PMC13387539; doi:10.1371/journal.pgph.0006913)
Supplement: Peer review history

# Author comment: Author response to Decision Letter 0

**DOI:** 10.1371/journal.pgph.0006913.r001

10 Mar 2026

Attachment Submitted filename: Response to editor comments.pdf

# Peer review: Decision Letter 0

**DOI:** 10.1371/journal.pgph.0006913.r002

**Authors:** Damen Haile Mariam

7 Apr 2026

PGPH-D-26-00831

Khat Consumers Exposure to Toxicity: Agrochemical Toxicity Awareness, Protective Practice and Self-Reported Health Ailments Among Khat Consumers in Ethiopia

PLOS Global Public Health

Dear Dr. Hassen,

Thank you for submitting your manuscript to PLOS Global Public Health. After careful consideration, we feel that it has merit but does not fully meet PLOS Global Public Health’s publication criteria as it currently stands. Therefore, we invite you to submit a revised version of the manuscript that addresses the points raised during the review process.

Please submit your revised manuscript by May 22 2026 11:59PM. If you will need more time than this to complete your revisions, please reply to this message or contact the journal office at globalpubhealth@plos.org. When you're ready to submit your revision, log on to https://www.editorialmanager.com/pgph/ and select the 'Submissions Needing Revision' folder to locate your manuscript file.

Please include the following items when submitting your revised manuscript:

- A letter that responds to each point raised by the editor and reviewer(s). You should upload this letter as a separate file labeled 'Response to Reviewers'.
- A marked-up copy of your manuscript that highlights changes made to the original version. You should upload this as a separate file labeled 'Revised Manuscript with Track Changes'.
- An unmarked version of your revised paper without tracked changes. You should upload this as a separate file labeled 'Manuscript'.

Guidelines for resubmitting your figure files are available below the reviewer comments at the end of this letter.

As the corresponding author, your ORCID iD is verified in the submission system and will appear in the published article. PLOS supports the use of ORCID, and we encourage all coauthors to register for an ORCID iD and use it as well. Please encourage your coauthors to verify their ORCID iD within the submission system before final acceptance, as unverified ORCID iDs will not appear in the published article. *Only*  the individual author can complete the verification step; PLOS staff *cannot*  verify ORCID iDs on behalf of authors.

We look forward to receiving your revised manuscript.

Kind regards,

Damen Haile Mariam, MD, MPH, PhD

Academic Editor

PLOS Global Public Health

Journal Requirements:

1. Please provide separate figure files in .tif or .eps format.

For more information about figure files please see our guidelines:  LINK

https://journals.plos.org/globalpublichealth/s/figures

https://journals.plos.org/globalpublichealth/s/figures#loc-file-requirements

2. Some material included in your submission may be copyrighted. According to PLOS’s copyright policy, authors who use figures or other material (e.g., graphics, clipart, maps) from another author or copyright holder must demonstrate or obtain permission to publish this material under the Creative Commons Attribution 4.0 International (CC BY 4.0) License used by PLOS journals. Please closely review the details of PLOS’s copyright requirements here: PLOS Licenses and Copyright. If you need to request permissions from a copyright holder, you may use PLOS's Copyright Content Permission form.

Please respond directly to this email or email the journal office and provide any known details concerning your material's license terms and permissions required for reuse, even if you have not yet obtained copyright permissions or are unsure of your material's copyright compatibility.

Potential Copyright Issues:

Figure 1: please (a) provide a direct link to the base layer of the map (i.e., the country or region border shape) and ensure this is also included in the figure legend; and (b) provide a link to the terms of use / license information for the base layer image or shapefile. We cannot publish proprietary or copyrighted maps (e.g. Google Maps, Mapquest) and the terms of use for your map base layer must be compatible with our CC-BY 4.0 license.

Note: if you created the map in a software program like R or ArcGIS, please locate and indicate the source of the basemap shapefile onto which data has been plotted.

If your map was obtained from a copyrighted source please amend the figure so that the base map used is from an openly available source. Alternatively, please provide explicit written permission from the copyright holder granting you the right to publish the material under our CC-BY 4.0 license.

Please note that the following CC BY licenses are compatible with PLOS license: CC BY 4.0, CC BY 2.0 and CC BY 3.0, meanwhile such licenses as CC BY-ND 3.0 and others are not compatible due to additional restrictions.

If you are unsure whether you can use a map or not, please do reach out and we will be able to help you. The following websites are good examples of where you can source open access or public domain maps:

* U.S. Geological Survey (USGS) - All maps are in the public domain. (http://www.usgs.gov)

* PlaniGlobe - All maps are published under a Creative Commons license so please cite “PlaniGlobe, http://www.planiglobe.com, CC BY 2.0” in the image credit after the caption. (http://www.planiglobe.com/?lang=enl)

* Natural Earth - All maps are public domain. (http://www.naturalearthdata.com/about/terms-of-use/)

Figure 2: Please confirm whether you drew the images / clip-art within the figure panels by hand. If you did not draw the images, please provide (a) a link to the source of the images or icons and their license / terms of use; or (b) written permission from the copyright holder to publish the images or icons under our CC-BY 4.0 license. Alternatively, you may replace the images with open source alternatives. See these open source resources you may use to replace images / clip-art:

- https://commons.wikimedia.org

- https://openclipart.org/

If the reviewer comments include a recommendation to cite specific previously published works, please review and evaluate these publications to determine whether they are relevant and should be cited. There is no requirement to cite these works unless the editor has indicated otherwise.

Additional Editor Comments (if provided):

Reviewer 1 -

- General Comment:

- This manuscript addresses an important public health issue in Ethiopia.

- However, the study suffers from serious methodological flaws that compromise the validity and clarity of the findings.

- The design is poorly articulated (cross-sectional vs. mixed methods), the application of the “One Health” lens is unjustified, the data collection and analysis procedures are inconsistently described, and the results are presented in a confusing

manner with excessive tables and unclear distinctions between quantitative and qualitative components.

- The discussion does not adequately interpret the findings in light of the methodological limitations. A major revision is required to clarify the study design, align methods with objectives, streamline results, and provide a coherent discussion.

- Specific comments are outlined below.

- Abstract part:

- Background: well written, the problem and gap are indicated. However, the objectives need to be rewritten because the word “explored” cannot be used for a quantitative study. Instead, use “assess” since the study design is a cross-sectional

study.

- Methods part: there is some confusion.

- “Utilizing a One Health lens, a cross-sectional study was conducted in Dire Dawa, Harar, and Jigjiga with 803 regular khat chewers. Data from semi structured interviews were analyzed via descriptive statistics, chi-square tests, and

multivariate binary logistic regression for quantitative components, whereas qualitative data were subjected to inductive thematic analysis to provide rich contextual detail regarding lived experiences.” This paragraph is very confused.

- i. The study design: is it only a cross-sectional study or a mixed method? If mixed, what type of mixed (sequential, concurrent, etc.)?

- ii. What is “Utilizing a One Health lens”? If the study is utilizing a One Health approach, it should include human, animal, and environment, but the study is conducted only by interviewing people who experience khat chewing.

- iii. “Semi structured interviews were analyzed via descriptive statistics, chi-square tests, and multivariate binary logistic regression for quantitative components” – how can semi-structured interviews be used for quantitative analysis?

Semi-structured interviews are for qualitative study.

- Results part: the paper seems to have two objectives:

- 1. To assess awareness and protective practices regarding the potential of multiple toxins and assessed related self-reported ailments among adult chewers to inform multisectoral reform.

- 2. To explore lived experiences. But the results only talk about awareness and protective practices even without CI of the level.

- Conclusion: I did not see any finding which supports the recommendation of “One Health approach; integrating agricultural reform, environmental monitoring, and public health to address systemic risks and dismantle structural barriers beyond

individual behavior.”

- 2. Introduction

- First paragraph: merge the citations [2]–[4].

- Paragraph 4: some statements are italic – make it uniform.

- Please summarize the introduction and health effects of khat into one paragraph and move to your main objective (agrochemical toxicity awareness, protective practice, and self-reported health ailments – the effect of agrochemical toxicity on

health, then the awareness of the khat chewers, protective practice and self-reported health ailments, then the gap, and lastly how to fill the gap – your objective).

- “The foundation of this study’s approach to predicting protective behavior is the theory of planned behavior (TPB)”; this part should be removed from the introduction. You can give a brief explanation of the theory used in the methods part.

- 3. Methods

- Study design and area: No need for elaboration of the design (“This approach allowed for the statistical identification of associations between variables and provided rich contextual detail regarding the chewers’ perceptions and lived experiences

of toxicity”) – please remove this.

- Study population, inclusion and exclusion are not well indicated.

- Study variables with operational definitions: not clear and not cited.

- Sample size determination, sampling method: it talks about quantitative study and is not clearly indicated. Why 50%? Why design effect? How random selection?

- Data collection tools and procedure: confusing. The data collection method is in-depth interview, which clearly indicates a qualitative study, but the tool is for quantitative.

- Qualitative analysis: indicated, but it is not clear from where, from whom, and how these data were generated.

- Data quality assurance: also talks only about quantitative study.

- All in all, the methods part is poorly written and not articulated with the objectives.

- 4. Results

- The authors make the results very complicated. For example, in the sociodemographic section, it is preferable to show clear sociodemographic factors; no need to classify with “aware” and “not aware”. Please check this again.

- Tables 2, 3, 4, 5: the variables are directly copied from the questionnaires without any amendment. For example, “How often do you chew khat?” could be “frequency of chewing per week” etc. All findings in the table have textual explanation

which is not recommendable; the reader also finds it hard to read. Focus on important findings, then the reader can see the other findings from the table.

- Section 3.1.4. Protective Practices and Barriers: what composes the level of protective practices? And why do you talk about awareness under this heading?

- Table 6: how did you assess anxiety, depression, sexual/reproductive health (decreased libido, premature ejaculation, erectile dysfunction)? How did you assess these sensitive issues?

- Table 6: is it multivariate or multivariable? Not clear. Please include all variables with p-value <0.2 in the model and indicate all descriptions of the result, including reference category. For example, “Weekly (vs. Daily)” – p-value is significant

while CI is not, so please check.

- Table 7: It is also the same. There is too much information in the results part: five descriptive statistics tables and two inferential statistics tables, which is too much. Please select important variables only.

- No findings of qualitative study.

- 5. Discussion

- The discussion is confusing.

Reviewer 2 -

- General comments

- The paper presents an interesting discussion on khat use and the potential exposure to DDT toxicity, both of which may have important health implications.

- However, it is unclear whether all khat is actually sprayed with DDT, as some farmers may not use this chemical. This raises an essential question: are the health issues described by the authors attributable to khat itself, to DDT contamination,

or to a combination of both? This ambiguity makes the focus of the paper somewhat unclear, leaving readers uncertain as to whether the primary concern is khat consumption or pesticide exposure.

- Overall, the manuscript would benefit from substantial revisions to improve clarity, strengthen its structure, and enhance methodological transparency.

- Introduction

- The introduction is too long and difficult to follow, largely due to a lack of logical flow. Some paragraphs appear more appropriate for the methodology section rather than the introduction. There are also inconsistencies in font style and

noticeable repetition of ideas.

- The introduction should focus on clearly defining khat, as the authors have attempted, along with describing the chemicals used to spray it, levels of awareness among users and farmers, and the potential effects on wellbeing, including

economic and social consequences. It should also summarize what is already known, identify gaps in existing knowledge, and explain the specific contribution of this paper.

- Additionally, the last paragraph on page 4 and the first two paragraphs on page 5 belong in the methodological section, not the introduction.

- Methods:

- Sampling

- It is not clear how the khat retailers were selected. Were all khat retailers in each town first identified and listed, and then randomly selected from that sampling frame? Additionally, the selection process may have implications for the findings.

For example, retailers selling more expensive khat may be more likely to source products that have been sprayed with pesticides such as DDT, whereas retailers selling cheaper khat may be dealing with products contaminated with other

substances such as dust or dirt. Clarifying the sampling strategy is essential to understand whether these differences were accounted for. Additionally, it is unclear whether interviews were conducted at the retailers' premises, which may be

challenging since khat chewers often rush to their preferred chewing locations and may not be willing to stay for an interview.

- Study variables

- Awareness, which is the outcome variable, is not clearly defined. It is unclear what specific domains or components were used to measure awareness, and whether a standardized or previously validated framework was applied. If the authors

adapted an existing tool or definition, the source should be clearly cited. The paper should explicitly describe how awareness was measured, including the indicators, scoring method, and criteria used to determine levels of awareness.

'

- Data analysis

- The manuscript does not specify which software was used to analyze the quantitative and qualitative data. Referring only to “appropriate software” is unclear and should be revised. The authors need to explicitly state the software packages

used (e.g., STATA, SPSS, R for quantitative data; NVivo, ATLAS.ti, or manual thematic coding for qualitative data).

- Additionally, the qualitative section appears shallow and lacks methodological detail. The authors should describe the qualitative analysis process more thoroughly, including how transcripts were coded, whether a thematic or content analysis

approach was used, how coding reliability was ensured, and any steps taken to enhance trustworthiness (e.g., triangulation, member checking).

- Results:

- Some parts of the results are not presented in logical order. For instance, the authors begin by presenting awareness levels without first describing the socio demographic characteristics of the study participants. Typically, results should start

with the demographic profile to contextualize subsequent findings.

- Regarding Table 4, the authors should clarify the distinction between the questions “Do you take steps to reduce your exposure to potential toxins in khat?” and “Do you take precautions to reduce the health risks of chewing khat?” The two

questions appear similar, yet they focus on different aspects. Additionally, the first question, which is more relevant to the paper’s core topic (exposure to contaminants), does not include any follow-up asking what specific steps participants

take to reduce toxin exposure. Meanwhile, the second question includes follow-up details, creating an imbalance in the information provided.

- The authors report the prevalence of self-reported ailments attributed to contaminated khat chewing, but these ailments could also arise from chewing uncontaminated khat. The results should therefore be interpreted and presented with

caution. This limitation, namely, the inability to determine whether symptoms are due to khat itself, contaminants like DDT, or a combination of both, should be explicitly acknowledged.

- The list of ailments assessed in the study is not exhaustive. Important health conditions potentially associated with pesticide exposure, such as symptoms of hepatitis, carcinomas were not included. The omission of these key health outcomes

limits the ability to fully understand the spectrum of possible effects and should be acknowledged as a limitation.

- In Tables 6 and 7, some odds ratios have very wide confidence intervals. The authors should re-check their data and model specification, as wide CIs may indicate issues such as small cell counts, insufficient sample size, or unstable estimates.

- Discussion:

- The discussion section needs to be strengthened to provide a more comprehensive synthesis of the findings. It should compare the results of this study with similar studies, highlighting consistencies, differences, and possible explanations.

- The discussion should also interpret what the findings imply for health, economic, and social wellbeing, especially in the context of khat use and potential exposure to contaminants such as DDT.

- The authors should reflect on current efforts aimed at reducing the harmful effects associated with khat production and consumption. This includes discussing existing regulatory mechanisms, community awareness programs, or agricultural

practices. It is also important to address the policy dilemma faced by the government, given that khat contributes substantial tax revenue. The discussion should explore what actions are feasible, what challenges exist, and what interventions

may not be realistic under current conditions.

- Additionally, it would be helpful to elaborate on the agricultural dimension, specifically, why farmers prefer cultivating khat as a cash crop instead of engaging in food crop production, and how this shift may negatively affect the food security

outlook in khat producing areas. The authors should also describe what measures, if any, are currently in place to reduce the use of DDT and other harmful pesticides in khat farming.

- Figure 2 should be placed in the methods section, as it represents a conceptual framework and aligns more appropriately with the methodological description of the study.

- The authors have not acknowledged any limitations. As noted in my comments under the results section, the study cannot determine whether reported ailments are caused by khat itself, by contaminants such as DDT, or by a combination of

both. Other methodological limitations, such as sampling, measurement biases, and wide confidence intervals—should be explicitly acknowledged.

[Note: HTML markup is below. Please do not edit.]

Reviewers' comments:

Reviewer's Responses to Questions

**Comments to the Author**

1. Does this manuscript meet PLOS Global Public Health’s publication criteria? Is the manuscript technically sound, and do the data support the conclusions? The manuscript must describe methodologically and ethically rigorous research with conclusions that are appropriately drawn based on the data presented.

Reviewer #1: Yes

Reviewer #2: Yes

**********

2. Has the statistical analysis been performed appropriately and rigorously?

Reviewer #1: No

Reviewer #2: Yes

**********

3. Have the authors made all data underlying the findings in their manuscript fully available (please refer to the Data Availability Statement at the start of the manuscript PDF file)?

The PLOS Data policy requires authors to make all data underlying the findings described in their manuscript fully available without restriction, with rare exception. The data should be provided as part of the manuscript or its supporting information, or deposited to a public repository. For example, in addition to summary statistics, the data points behind means, medians and variance measures should be available. If there are restrictions on publicly sharing data—e.g. participant privacy or use of data from a third party—those must be specified.

Reviewer #1: Yes

Reviewer #2: No

**********

4. Is the manuscript presented in an intelligible fashion and written in standard English?

PLOS Global Public Health does not copyedit accepted manuscripts, so the language in submitted articles must be clear, correct, and unambiguous. Any typographical or grammatical errors should be corrected at revision, so please note any specific errors here.

Reviewer #1: No

Reviewer #2: No

**********

5. Review Comments to the Author

Please use the space provided to explain your answers to the questions above. You may also include additional comments for the author, including concerns about dual publication, research ethics, or publication ethics. (Please upload your review as an attachment if it exceeds 20,000 characters)

Reviewer #1: Revised General Comment

This manuscript addresses an important public health issue in Ethiopia. However, the study suffers from serious methodological flaws that compromise the validity and clarity of the findings. The design is poorly articulated (cross-sectional vs. mixed methods), the application of the “One Health” lens is unjustified, the data collection and analysis procedures are inconsistently described, and the results are presented in a confusing manner with excessive tables and unclear distinctions between quantitative and qualitative components. The discussion does not adequately interpret the findings in light of the methodological limitations. A major revision is required to clarify the study design, align methods with objectives, streamline results, and provide a coherent discussion. Specific comments are outlined below

1. Abstract part

Background: well written, the problem and gap are indicated.However, the objectives need to be rewritten because the word “explored” cannot be used for a quantitative study. Instead, use “assess” since the study design is a cross-sectional study.

Method part: there is some confusion.

• “Utilizing a One Health lens, a cross-sectional study was conducted in Dire Dawa, Harar, and Jigjiga with 803 regular khat chewers. Data from semi structured interviews were analyzed via descriptive statistics, chi-square tests, and multivariate binary logistic regression for quantitative components, whereas qualitative data were subjected to inductive thematic analysis to provide rich contextual detail regarding lived experiences.” This paragraph is very confused.

i. The study design: is it only a cross-sectional study or a mixed method? If mixed, what type of mixed (sequential, concurrent, etc.)?

ii. What is “Utilizing a One Health lens”? If the study is utilizing a One Health approach, it should include human, animal, and environment, but the study is conducted only by interviewing people who experience khat chewing.

iii. “Semi structured interviews were analyzed via descriptive statistics, chi-square tests, and multivariate binary logistic regression for quantitative components” – how can semi-structured interviews be used for quantitative analysis? Semi-structured interviews are for qualitative study.

Result part: the paper seems to have two objectives:

1. To assess awareness and protective practices regarding the potential of multiple toxins and assessed related self-reported ailments among adult chewers to inform multisectoral reform.

2. To explore lived experiences. But the results only talk about awareness and protective practices even without CI of the level.

Conclusion: I did not see any finding which supports the recommendation of “One Health approach; integrating agricultural reform, environmental monitoring, and public health to address systemic risks and dismantle structural barriers beyond individual behavior.”

2. Introduction

• First paragraph: merge the citations [2]–[4].

• Paragraph 4: some statements are italic – make it uniform.

• Please summarize the introduction and health effects of khat into one paragraph and move to your main objective (agrochemical toxicity awareness, protective practice, and self-reported health ailments – the effect of agrochemical toxicity on health, then the awareness of the khat chewers, protective practice and self-reported health ailments, then the gap, and lastly how to fill the gap – your objective).

• “The foundation of this study’s approach to predicting protective behavior is the theory of planned behavior (TPB)”; this part should be removed from the introduction. You can give a brief explanation of the theory used in the methods part.

3. Methods

Study design and area: No need for elaboration of the design (“This approach allowed for the statistical identification of associations between variables and provided rich contextual detail regarding the chewers’ perceptions and lived experiences of toxicity”) – please remove this.

• Study population, inclusion and exclusion are not well indicated.

• Study variables with operational definitions: not clear and not cited.

• Sample size determination, sampling method: it talks about quantitative study and is not clearly indicated. Why 50%? Why design effect? How random selection?

• Data collection tools and procedure: confusing. The data collection method is in-depth interview, which clearly indicates a qualitative study, but the tool is for quantitative.

• Qualitative analysis: indicated, but it is not clear from where, from whom, and how these data were generated.

• Data quality assurance: also talks only about quantitative study.

All in all, the methods part is poorly written and not articulated with the objectives.

4. Results

• The authors make the results very complicated. For example, in the sociodemographic section, it is preferable to show clear sociodemographic factors; no need to classify with “aware” and “not aware”. Please check this again.

• Tables 2, 3, 4, 5: the variables are directly copied from the questionnaires without any amendment. For example, “How often do you chew khat?” could be “frequency of chewing per week” etc. All findings in the table have textual explanation which is not recommendable; the reader also finds it hard to read. Focus on important findings, then the reader can see the other findings from the table.

• Section 3.1.4. Protective Practices and Barriers: what composes the level of protective practices? And why do you talk about awareness under this heading?

• Table 6: how did you assess anxiety, depression, sexual/reproductive health (decreased libido, premature ejaculation, erectile dysfunction)? How did you assess these sensitive issues?

• Table 6: is it multivariate or multivariable? Not clear. Please include all variables with p-value <0.2 in the model and indicate all descriptions of the result, including reference category. For example, “Weekly (vs. Daily)” – p-value is significant while CI is not, so please check.

• Table 7: also the same. There is too much information in the results part: five descriptive statistics tables and two inferential statistics tables, which is too much. Please select important variables only.

• No findings of qualitative study.

5. Discussion

The discussion is confusing.

Reviewer #2: General comments

The paper presents an interesting discussion on khat use and the potential exposure to DDT toxicity, both of which may have important health implications. However, it is unclear whether all khat is actually sprayed with DDT, as some farmers may not use this chemical. This raises an essential question: are the health issues described by the authors attributable to khat itself, to DDT contamination, or to a combination of both? This ambiguity makes the focus of the paper somewhat unclear, leaving readers uncertain as to whether the primary concern is khat consumption or pesticide exposure. Overall, the manuscript would benefit from substantial revisions to improve clarity, strengthen its structure, and enhance methodological transparency.

Introduction

The introduction is too long and difficult to follow, largely due to a lack of logical flow. Some paragraphs appear more appropriate for the methodology section rather than the introduction. There are also inconsistencies in font style and noticeable repetition of ideas.

The introduction should focus on clearly defining khat, as the authors have attempted, along with describing the chemicals used to spray it, levels of awareness among users and farmers, and the potential effects on wellbeing, including economic and social consequences. It should also summarize what is already known, identify gaps in existing knowledge, and explain the specific contribution of this paper.

Additionally, the last paragraph on page 4 and the first two paragraphs on page 5 belong in the methodological section, not the introduction.

Methods

Sampling

It is not clear how the khat retailers were selected. Were all khat retailers in each town first identified and listed, and then randomly selected from that sampling frame? Additionally, the selection process may have implications for the findings. For example, retailers selling more expensive khat may be more likely to source products that have been sprayed with pesticides such as DDT, whereas retailers selling cheaper khat may be dealing with products contaminated with other substances such as dust or dirt. Clarifying the sampling strategy is essential to understand whether these differences were accounted for. Additionally, it is unclear whether interviews were conducted at the retailers' premises, which may be challenging since khat chewers often rush to their preferred chewing locations and may not be willing to stay for an interview.

Study variables

Awareness, which is the outcome variable, is not clearly defined. It is unclear what specific domains or components were used to measure awareness, and whether a standardized or previously validated framework was applied. If the authors adapted an existing tool or definition, the source should be clearly cited. The paper should explicitly describe how awareness was measured, including the indicators, scoring method, and criteria used to determine levels of awareness.

Data analysis

The manuscript does not specify which software was used to analyze the quantitative and qualitative data. Referring only to “appropriate software” is unclear and should be revised. The authors need to explicitly state the software packages used (e.g., STATA, SPSS, R for quantitative data; NVivo, ATLAS.ti, or manual thematic coding for qualitative data).

Additionally, the qualitative section appears shallow and lacks methodological detail. The authors should describe the qualitative analysis process more thoroughly, including how transcripts were coded, whether a thematic or content analysis approach was used, how coding reliability was ensured, and any steps taken to enhance trustworthiness (e.g., triangulation, member checking).

Result

Some parts of the results are not presented in logical order. For instance, the authors begin by presenting awareness levels without first describing the socio‑demographic characteristics of the study participants. Typically, results should start with the demographic profile to contextualize subsequent findings.

Regarding Table 4, the authors should clarify the distinction between the questions “Do you take steps to reduce your exposure to potential toxins in khat?” and “Do you take precautions to reduce the health risks of chewing khat?” The two questions appear similar, yet they focus on different aspects. Additionally, the first question, which is more relevant to the paper’s core topic (exposure to contaminants), does not include any follow-up asking what specific steps participants take to reduce toxin exposure. Meanwhile, the second question includes follow-up details, creating an imbalance in the information provided.

The authors report the Prevalence of Self‑Reported Ailments Attributed to Contaminated Khat Chewing, but these ailments could also arise from chewing uncontaminated khat. The results should therefore be interpreted and presented with caution. This limitation, namely, the inability to determine whether symptoms are due to khat itself, contaminants like DDT, or a combination of both, should be explicitly acknowledged.

The list of ailments assessed in the study is not exhaustive. Important health conditions potentially associated with pesticide exposure, such as symptoms of hepatitis, carcinomas were not included. The omission of these key health outcomes limits the ability to fully understand the spectrum of possible effects and should be acknowledged as a limitation.

In Tables 6 and 7, some odds ratios have very wide confidence intervals. The authors should re-check their data and model specification, as wide CIs may indicate issues such as small cell counts, insufficient sample size, or unstable estimates.

Discussion

The discussion section needs to be strengthened to provide a more comprehensive synthesis of the findings. It should compare the results of this study with similar studies, highlighting consistencies, differences, and possible explanations. The discussion should also interpret what the findings imply for health, economic, and social wellbeing, especially in the context of khat use and potential exposure to contaminants such as DDT.

The authors should reflect on current efforts aimed at reducing the harmful effects associated with khat production and consumption. This includes discussing existing regulatory mechanisms, community awareness programs, or agricultural practices. It is also important to address the policy dilemma faced by the government, given that khat contributes substantial tax revenue. The discussion should explore what actions are feasible, what challenges exist, and what interventions may not be realistic under current conditions.

Additionally, it would be helpful to elaborate on the agricultural dimension, specifically, why farmers prefer cultivating khat as a cash crop instead of engaging in food crop production, and how this shift may negatively affect the food security outlook in khat‑producing areas. The authors should also describe what measures, if any, are currently in place to reduce the use of DDT and other harmful pesticides in khat farming.

Figure 2 should be placed in the methods section, as it represents a conceptual framework and aligns more appropriately with the methodological description of the study.

The authors have not acknowledged any limitations. As noted in my comments under the results section, the study cannot determine whether reported ailments are caused by khat itself, by contaminants such as DDT, or by a combination of both. Other methodological limitations, such as sampling, measurement biases, and wide confidence intervals—should be explicitly acknowledged.

**********

6. PLOS authors have the option to publish the peer review history of their article (what does this mean?). If published, this will include your full peer review and any attached files.

**Do you want your identity to be public for this peer review?**  If you choose “no”, your identity will remain anonymous but your review may still be made public.

For information about this choice, including consent withdrawal, please see our Privacy Policy.

Reviewer #1: No

Reviewer #2: No

**********

[NOTE: If reviewer comments were submitted as an attachment file, they will be attached to this email and accessible via the submission site. Please log into your account, locate the manuscript record, and check for the action link "View Attachments". If this link does not appear, there are no attachment files.]

-->Figure Resubmissions:

While revising your submission, we strongly recommend that you use PLOS’s NAAS tool (https://ngplosjournals.pagemajik.ai/artanalysis) to test your figure files. NAAS can convert your figure files to the TIFF file type and meet basic requirements (such as print size, resolution), or provide you with a report on issues that do not meet our requirements and that NAAS cannot fix.

After uploading your figures to PLOS’s NAAS tool - https://ngplosjournals.pagemajik.ai/artanalysis, NAAS will process the files provided and display the results in the "Uploaded Files" section of the page as the processing is complete. If the uploaded figures meet our requirements (or NAAS is able to fix the files to meet our requirements), the figure will be marked as "fixed" above. If NAAS is unable to fix the files, a red "failed" label will appear above. When NAAS has confirmed that the figure files meet our requirements, please download the file via the download option, and include these NAAS processed figure files when submitting your revised manuscript.

Attachment Submitted filename: Comments PGPH.docx

# Author comment: Author response to Decision Letter 1

**DOI:** 10.1371/journal.pgph.0006913.r003

9 Jun 2026

Attachment Submitted filename: Point by point responses .pdf

# Editor report: Decision Letter 1

**DOI:** 10.1371/journal.pgph.0006913.r004

**Authors:** Damen Haile Mariam

25 Jun 2026

PGPH-D-26-00831R1

Khat Consumers Exposure to Toxicity: Agrochemical Toxicity Awareness, Protective Practice and Self-Reported Health Ailments Among Khat Consumers in Ethiopia

PLOS Global Public Health

Dear Dr. Hassen,

Thank you for submitting your manuscript to PLOS Global Public Health. After careful consideration, we feel that it has merit but does not fully meet PLOS Global Public Health’s publication criteria as it currently stands. Therefore, we invite you to submit a revised version of the manuscript that addresses the points raised during the review process.

Please submit your revised manuscript by Jul 25 2026 11:59PM. If you will need more time than this to complete your revisions, please reply to this message or contact the journal office at globalpubhealth@plos.org. When you're ready to submit your revision, log on to https://www.editorialmanager.com/pgph/ and select the 'Submissions Needing Revision' folder to locate your manuscript file.

Please include the following items when submitting your revised manuscript:

- A letter that responds to each point raised by the editor and reviewer(s). You should upload this letter as a separate file labeled 'Response to Reviewers'.
- A marked-up copy of your manuscript that highlights changes made to the original version. You should upload this as a separate file labeled 'Revised Manuscript with Track Changes'.
- An unmarked version of your revised paper without tracked changes. You should upload this as a separate file labeled 'Manuscript'.

Guidelines for resubmitting your figure files are available below the reviewer comments at the end of this letter.

As the corresponding author, your ORCID iD is verified in the submission system and will appear in the published article. PLOS supports the use of ORCID, and we encourage all coauthors to register for an ORCID iD and use it as well. Please encourage your coauthors to verify their ORCID iD within the submission system before final acceptance, as unverified ORCID iDs will not appear in the published article. *Only*  the individual author can complete the verification step; PLOS staff *cannot*  verify ORCID iDs on behalf of authors.

We look forward to receiving your revised manuscript.

Kind regards,

Damen Haile Mariam, MD, MPH, PhD

Academic Editor

PLOS Global Public Health

Journal Requirements:

1. Please provide separate main figure files in .tif or .eps format only and remove any figures embedded in your manuscript file. Please also ensure that all files are under our size limit of 10MB. Please leave the figure captions in the manuscript.

For more information about how to convert your figure files please see our guidelines: https://journals.plos.org/globalpublichealth/s/figures

2. We have noticed that you have uploaded Supporting Information files, but you have not included a list of legends. Please add a full list of legends for your Supporting Information files before or after the references list.

3. If the reviewer comments include a recommendation to cite specific previously published works, please review and evaluate these publications to determine whether they are relevant and should be cited. There is no requirement to cite these works unless the editor has indicated otherwise.

4. Please review your reference list to ensure that it is complete and correct. If you have cited papers that have been retracted, please include the rationale for doing so in the manuscript text, or remove these references and replace them with relevant current references. Any changes to the reference list should be mentioned in the rebuttal letter that accompanies your revised manuscript. If you need to cite a retracted article, indicate the article’s retracted status in the References list and also include a citation and full reference for the retraction notice.

Additional Editor Comments (if provided):

Minor Comment:

- The authors have substantially improved their manuscript and addressed most of the comments by the reviewers.

- However, they need to recheck their reference style. For instance, they cited as [1], [5]–[7], which should be [1, 5-7]. It should be in one set of brackets instead of separate ones.

[Note: HTML markup is below. Please do not edit.]

Reviewers' comments:

[NOTE: If reviewer comments were submitted as an attachment file, they will be attached to this email and accessible via the submission site. Please log into your account, locate the manuscript record, and check for the action link "View Attachments". If this link does not appear, there are no attachment files.]

Figure Resubmissions:

While revising your submission, we strongly recommend that you use PLOS’s NAAS tool (https://ngplosjournals.pagemajik.ai/artanalysis) to test your figure files. NAAS can convert your figure files to the TIFF file type and meet basic requirements (such as print size, resolution), or provide you with a report on issues that do not meet our requirements and that NAAS cannot fix.

After uploading your figures to PLOS’s NAAS tool - https://ngplosjournals.pagemajik.ai/artanalysis, NAAS will process the files provided and display the results in the "Uploaded Files" section of the page as the processing is complete. If the uploaded figures meet our requirements (or NAAS is able to fix the files to meet our requirements), the figure will be marked as "fixed" above. If NAAS is unable to fix the files, a red "failed" label will appear above. When NAAS has confirmed that the figure files meet our requirements, please download the file via the download option, and include these NAAS processed figure files when submitting your revised manuscript.

# Author comment: Author response to Decision Letter 2

**DOI:** 10.1371/journal.pgph.0006913.r005

1 Jul 2026

Attachment Submitted filename: Response to Reviewers .pdf

# Editor report: Decision Letter 2

**DOI:** 10.1371/journal.pgph.0006913.r006

**Authors:** Damen Haile Mariam

5 Jul 2026

Khat Consumers Exposure to Toxicity: Agrochemical Toxicity Awareness, Protective Practice and Self-Reported Health Ailments Among Khat Consumers in Ethiopia

PGPH-D-26-00831R2

Dear Dr. Hassen,

We are pleased to inform you that your manuscript 'Khat Consumers Exposure to Toxicity: Agrochemical Toxicity Awareness, Protective Practice and Self-Reported Health Ailments Among Khat Consumers in Ethiopia' has been provisionally accepted for publication in PLOS Global Public Health.

Before your manuscript can be formally accepted you will need to complete some formatting changes, which you will receive in a follow up email. A member of our team will be in touch with a set of requests.

Please note that your manuscript will not be scheduled for publication until you have made the required changes, so a swift response is appreciated.

IMPORTANT: The editorial review process is now complete. PLOS will only permit corrections to spelling, formatting or significant scientific errors from this point onwards. Requests for major changes, or any which affect the scientific understanding of your work, will cause delays to the publication date of your manuscript.

If your institution or institutions have a press office, please notify them about your upcoming paper to help maximize its impact. If they'll be preparing press materials, please inform our press team as soon as possible -- no later than 48 hours after receiving the formal acceptance. Your manuscript will remain under strict press embargo until 2 pm Eastern Time on the date of publication. For more information, please contact globalpubhealth@plos.org.

Thank you again for supporting Open Access publishing; we are looking forward to publishing your work in PLOS Global Public Health.

Best regards,

Damen Haile Mariam, MD, MPH, PhD

Academic Editor

PLOS Global Public Health

***********************************************************

Reviewer Comments (if any, and for reference):
